# Supplementary material for: Light regulates alternative splicing outcomes via the TOR kinase pathway
Source: Cell Rep. Author manuscript; Available in PMC 2021 Oct 26. (PMC8547716; doi:10.1016/j.celrep.2021.109676)
Supplement: Supplementary Materials [file NIHMS1745235-supplement-Supplementary_Materials.zip › 1-s2.0-S2211124721011207-mmc1.pdf]

Cell Reports, Volume *36*

Supplemental information

Light regulates alternative splicing outcomes via the TOR kinase pathway

Stefan Riegler, Lucas Servi, M. Regina Scarpin, Micaela A. Godoy Herz, María G. Kubaczka, Peter Venhuizen, Christian Meyer, Jacob O. Brunkard, Maria Kalyna, Andrea Barta, and Ezequiel Petrillo

Figure S1

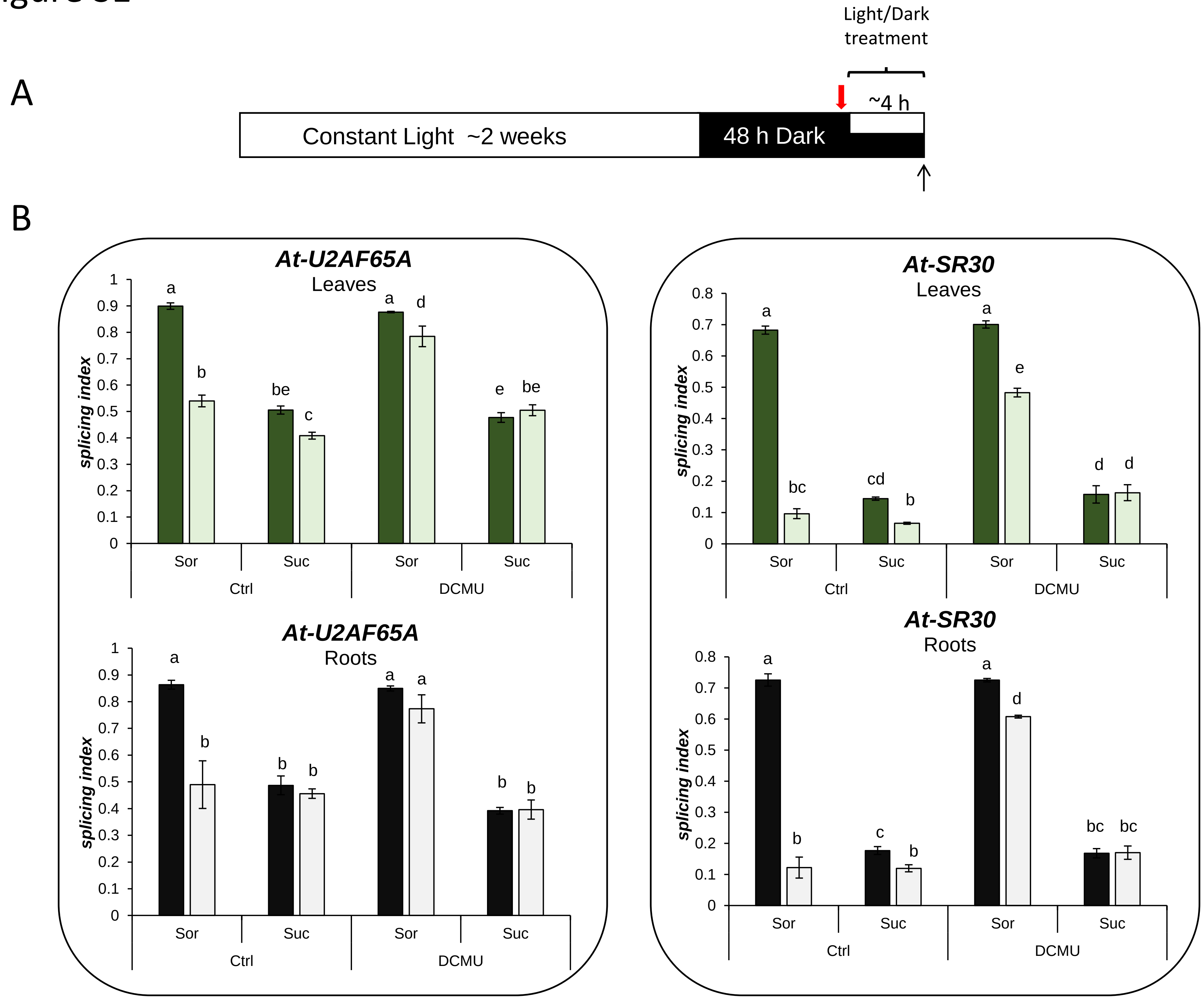

Figure S1. A) Standard treatment protocol. Seedlings were grown in Murashige and Skoog/2-(N-morpholino)ethanesulfonic acid buffered (MS-MES) agar plates (~15 seedlings per 10 cm Ø plate) for a period of two weeks and then transferred for 48 hours to darkness. After this period, seedlings were either transferred to light or kept in darkness for, approximately, additional four hours (light/dark treatment). Sorbitol, sucrose and the used drugs, were added in liquid media (20 mL) on top of the agar-growth media (20 mL) and vacuum was applied in order to ensure equal uptake by the different plant tissues. These compounds were added one hour before the end of the 48 hour darkness period (red arrow). Sample collection was performed at the end of the light/dark treatment and it is shown by the black arrow. When needed, prior sample collection, plants were dissected and leaves and roots were collected separatedly (post-treatment dissection). In particular experiments, plants were dissected before light/dark treatments (pre-treatment dissection). Related to Figures 1-3. **B) Sucrose effects on alternative splicing do not involve chloroplasts.** Alternative splicing changes are shown for *At-U2AF65A* (left) and *At-SR30* (right). *A. thaliana* seedlings were grown on MS-MES agar plates (~15 seeds per plate) for a period of two weeks under constant light, then transferred to darkness for 48 hours. Sorbitol (Sor, 100 mM) or sucrose (Suc, 100 mM) supplemented liquid media, with DCMU or without it (ethanol was used as control, Ctrl), were added on top of agar media one hour before the end of the 48 hours darkness period. Vacuum infiltration was applied for five minutes to increase the uptake of the different compounds by all the tissues. After the light (lighter bars) / dark (darker bars) treatments (~4h), leaves (green bars) and roots (grey bars) were dissected for sample collection. The graphs show splicing index means ± standard error (n=4). Same letters indicate means that are not statistically different (p>0.05). Statistics were done using InfoStat with Fisher LSD for comparisons. Related to Figures 1G-H.

Figure S2

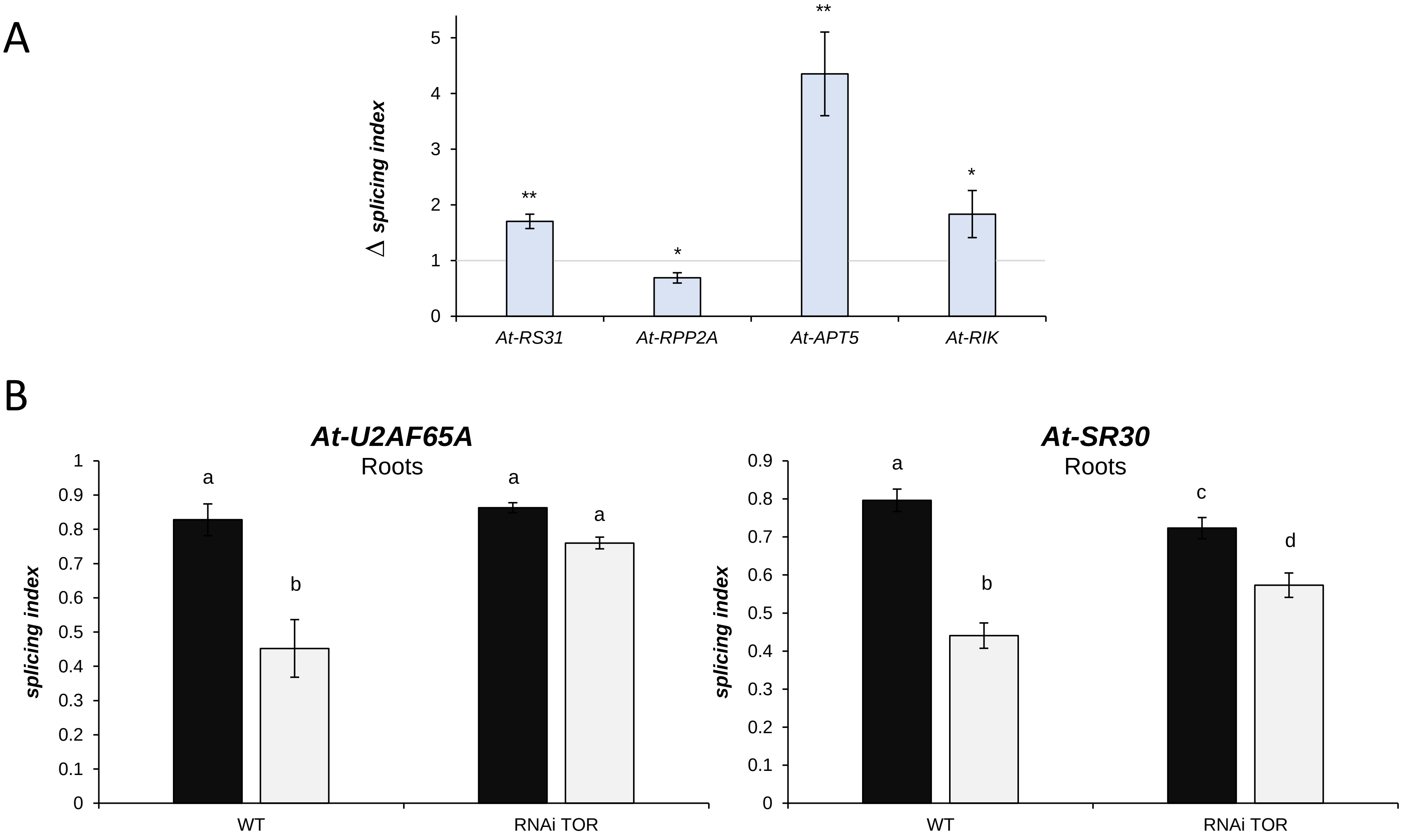

Figure S2. TOR regulates alternative splicing. A) Modulating TOR activity impacts alternative splicing of *At-RS31*, *RPP2A*, *APT5* and *RIK*. *A. thaliana* seedlings were grown on MS-MES agar plates (~15 seeds per plate) to quiescence and then supplied with either 15 mM glucose (control) or 15 mM glucose + 5 µM Torin2 (treatment). Following RT-PCR, splicing indexes were measured in both conditions and then related according to the following: Δ splicing index = splicing index (treatment) / splicing index (control). The graphs show Δ splicing index means ± standard deviation (n=3). Statistical significance was assessed via Student's t-test by comparing measured values with values drawn from normal distributions of mean = 1 (no change of splicing index upon treatment) with the same standard deviation as in the respective measured data. Student's t-test: ** P < 0.01; * P < 0.05. Related to Figure 2. **B) A transgenic RNAi knockdown line of TOR (RNAi TOR) shows disrupted responses to light on the splicing regulation in roots.** Alternative splicing changes are shown for *At-U2AF65A* and *At-SR30. A. thaliana* wild type (WT) and transgenic (35-7 TOR RNAi) line seedlings were grown on MS-MES agar plates (~15 seeds per plate) for a period of two weeks under constant light, then transferred to darkness for 48 hours. After light (lighter bars) / dark (darker bars) treatments for additional four hours, roots were dissected for sample collection. The graphs show splicing index means ± standard error (n=4). Same letters indicate means that are not statistically different (p>0.05). Statistical analyses were done using InfoStat with Fisher LSD for comparisons. Related to Figures 2B and 2D.

Figure S3

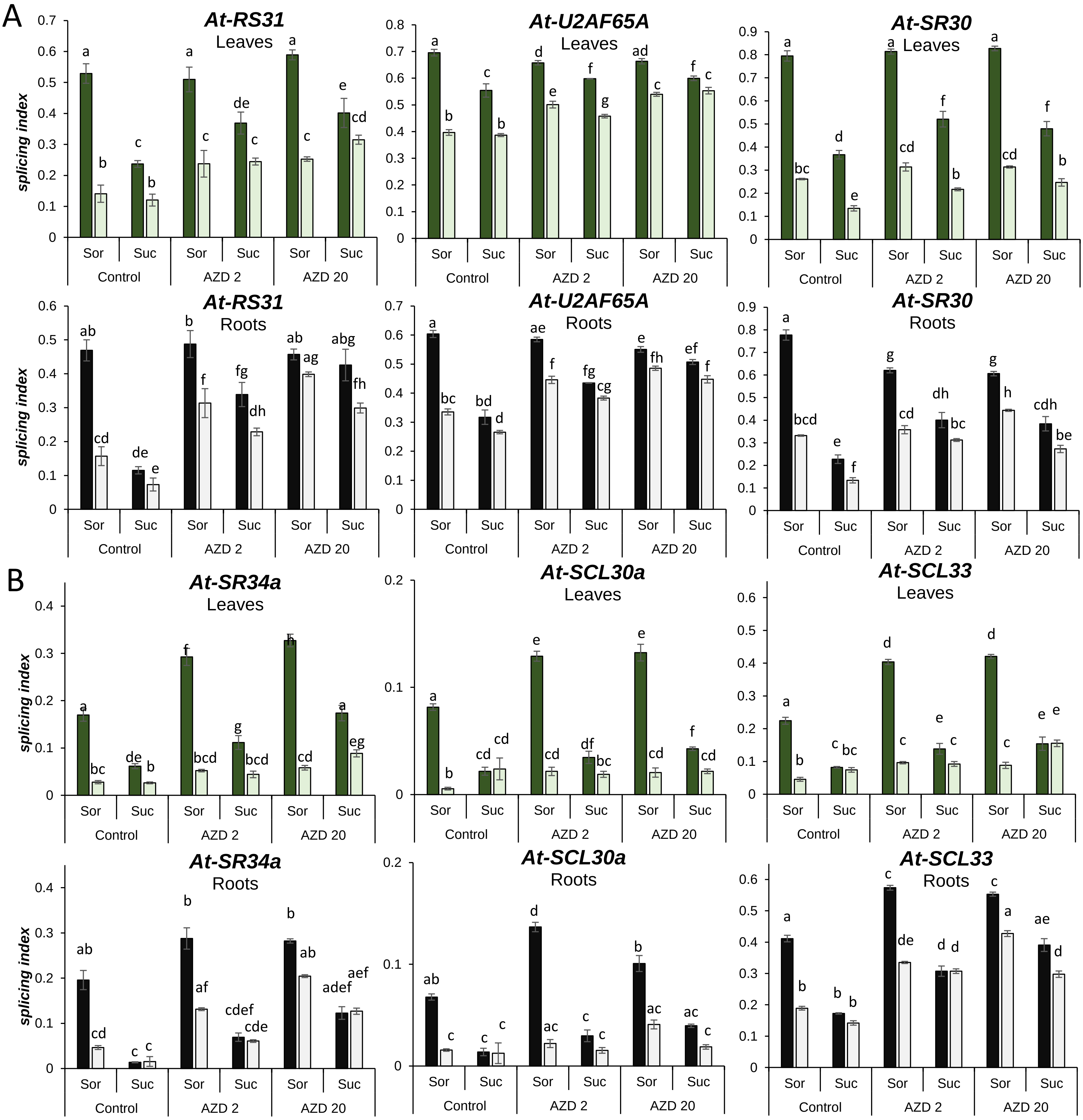

Figure S3: TOR kinase inhibition disrupts light and sucrose regulation of *At-RS31, At-U2AF65A, At-SR30* and *At-SR34a* but it has minor effects on *At-SCL30a* and *At-SCL33* alternative splicing events in roots. A-B) Alternative splicing changes are shown for *At-RS31* (**A,** left), *At-U2AF65A* (**A,** middle), *At-SR30* (**A,** right), *At-SR34a* (**B,** left), *At-SCL30a* (**B,** middle) and *At-SCL33* (**C,** right). Upper panels are leaves and bottom panels roots. *A. thaliana* seedlings were grown on MS-MES agar plates (~15 seeds per plate) for a period of two weeks under constant light, then transferred to darkness for 48 hours. Sorbitol (Sor, 100 mM) or sucrose (Suc, 100 mM) supplemented liquid media, with two concentrations of AZD-8055 2 µM (AZD 2) and 20 µM (AZD 20) or without it (DMSO was used as control, Ctrl). Compounds were added in solution on top of agar media one hour before the end of the 48 hours darkness period. Vacuum infiltration was applied for five minutes to increase the uptake by all the tissues. After the light (lighter bars) / dark (darker bars) treatments (~4h), leaves (green bars) and roots (grey bars) were dissected for sample collection. The graphs show splicing index means ± standard error (n=3). Same letters indicate means that are not statistically different (p>0.05). Statistics were done using InfoStat with Fisher LSD for comparisons. Related to Figures 2E-F.

Figure S4

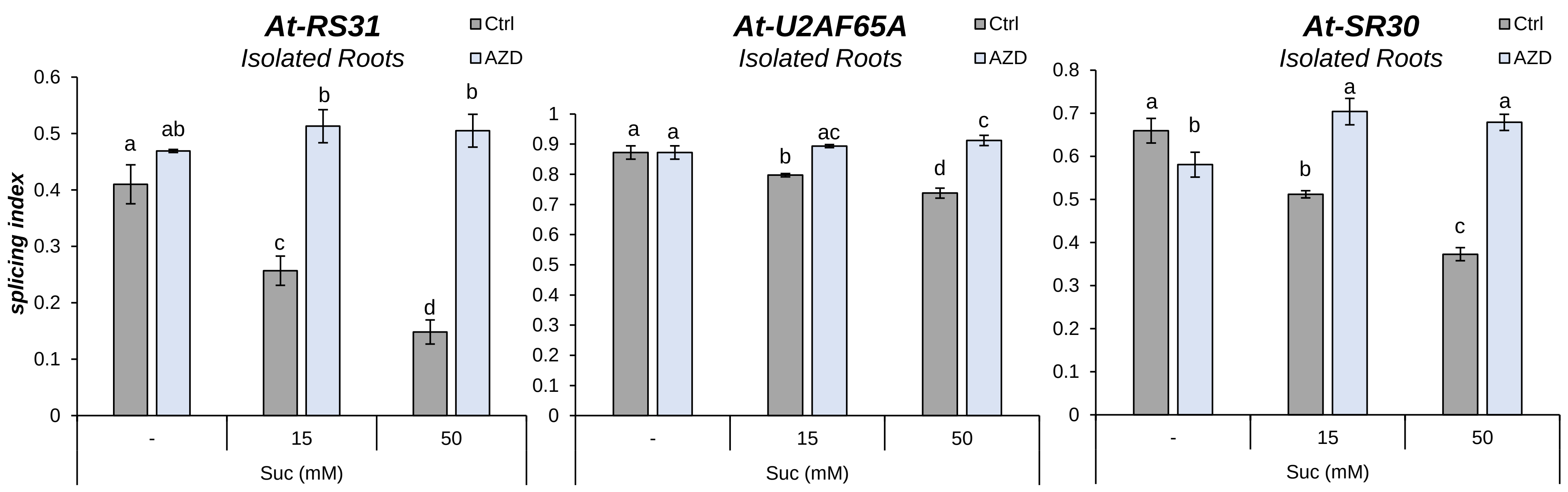

Figure S4. TOR kinase activity is necessary for isolated (detached) roots to change the alternative splicing of *At-RS31, At-U2AF65A* and *At-SR30* in response to sucrose. Sucrose (Suc) addition mimics light effects on alternative splicing, reducing the splicing index values in isolated roots in a dose dependent manner. The inhibition of TOR kinase activity by AZD-8055 (AZD) abolishes the effect of sucrose. *A. thaliana* plants were grown on MS-MES agar plates (~15 plants per plate) for a period of two weeks under constant light and then incubated in the dark for 48 hours. Roots were detached and transferred to 6-well plates with liquid media supplemented with sucrose 0, 15 or 50 mM. Sorbitol was used as osmotic control (to reach a total 50 mM concentration, together with sucrose, in every treatment). Twenty µM AZD-8055 (AZD, light blue bars) was used for treatments and dimethyl sulfoxide was used as control (Ctrl, grey bars). Vacuum was applied for five minutes to increase the uptake of the different compounds. The graphs show splicing index means ± standard error (n=3). Same letters indicate means that are not statistically different (p>0.05). Statistical analyses were done using InfoStat with Fisher LSD for comparisons. Related to Figures 2C-F.

Figure S5

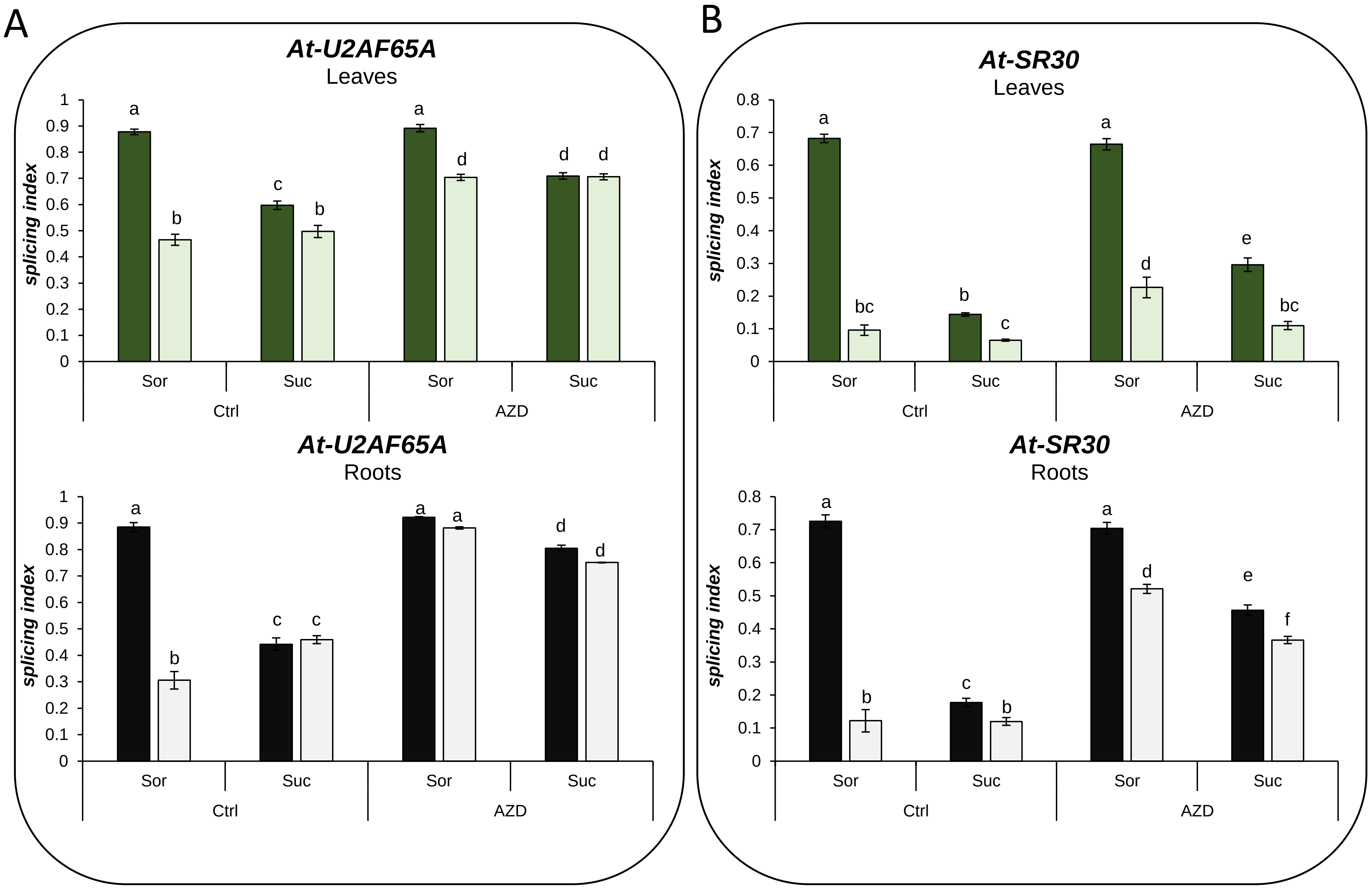

Figure S5. The inhibition of TOR kinase activity by AZD-8055 disrupts alternative splicing responses in roots. Alternative splicing changes are shown for (**A**) *At-U2AF65A* and (**B**) *At-SR30. A. thaliana* seedlings were grown on MS-MES agar plates (~15 seeds per plate) for a period of two weeks under constant light, then transferred to darkness for 48 hours. Sorbitol (Sor, 100 mM) or sucrose (Suc, 100 mM) supplemented liquid media, with 20 µM AZD-8055 (AZD) or without it (dimethyl sulfoxide as control, Ctrl), were added on top of the agar media one hour before the end of the 48 hours darkness period. Vacuum infiltration was applied for five minutes to increase the uptake of the different compounds by all the tissues. After the light (lighter bars) / dark (darker bars) treatments for additional four hours, leaves and roots were dissected for sample collection. The graphs show splicing index means ± standard error (n=4). Same letters indicate means that are not statistically different (p>0.05). Statistical analyses were done using InfoStat with Fisher LSD for comparisons. Related to Figures 2E-F, TOR kinase activity (RPS6-P/RPS6) can be seen there.

Figure S6

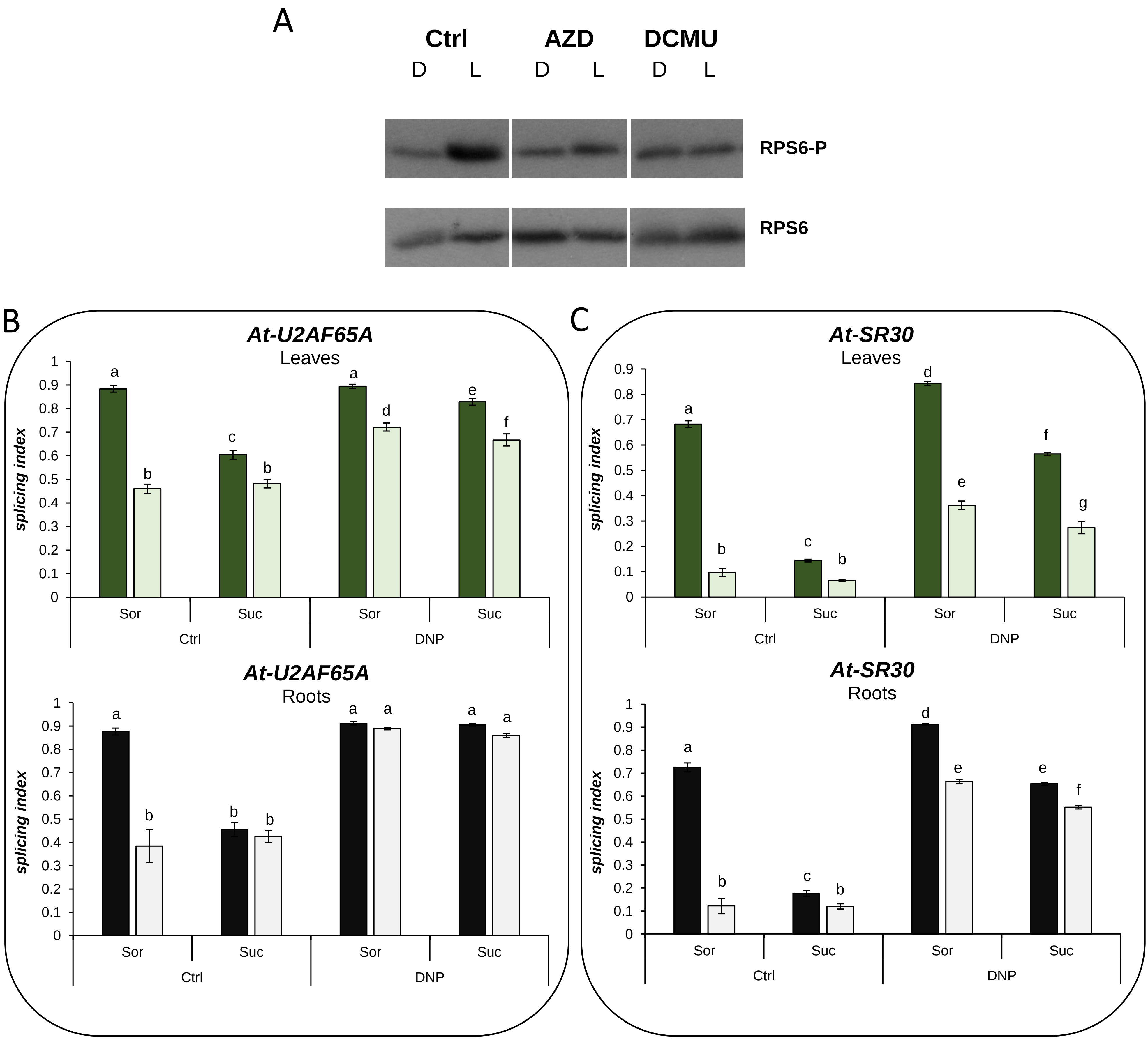

Figure S6. A) TOR kinase activity is modulated by the photosynthetic electron transport. Light (L) activates TOR kinase as revealed by the increase in the phosphorylation of RPS6 (RPS6-P) in light vs. dark (D). The inhibition of TOR kinase activity by AZD-8055 (AZD) diminishes the effect of light while blocking the electron transport with DCMU completely abolishes light induced phosphorylation of RPS6. Western blots to detect RPS6 phosphorylation and total levels in leaves after light/dark treatment in the presence of DCMU (15 µM) or AZD-8055 (20 µM). *A. thaliana* plants were grown on MS-MES agar plates (~15 plants per plate) for a period of two weeks under constant light and then incubated in the dark for 48 hours. Ethanol (for DCMU) and dimethyl sulfoxide (for AZD) were used combined as control (Ctrl) and supplemented to the same amounts in all the treatments. Vacuum was applied for five minutes to increase the uptake of the different compounds. Related to Figures 1G-H and 2C-D. **B-C) Proton gradients disruption by an uncoupler obliterates alternative splicing changes induced by light and sucrose in roots.** Alternative splicing changes are shown for (**B**) *At-U2AF65A* and (**C**) *At-SR30. A. thaliana* seedlings were grown on MS-MES agar plates (~15 seeds per plate) for a period of two weeks under constant light, then transferred to darkness for 48 hours. Sorbitol (Sor, 100 mM) or sucrose (Suc, 100 mM) supplemented media, with 20 µM DNP (dinitrophenol) or without it (ethanol was used as control, Ctrl), were added on top of the agar media one hour before the end of the 48 hours darkness period. Vacuum infiltration was applied for five minutes to increase the uptake of the different compounds by all the tissues. After light (lighter bars) / dark (darker bars) treatments for additional four hours, leaves and roots were dissected for sample collection. The graphs show splicing index means ± standard error (n=4). Same letters indicate means that are not statistically different (p>0.05). Statistical analyses were done using InfoStat with Fisher LSD for comparisons. Related to Figures 3A-B, TOR kinase activity (RPS6-P/RPS6) can be seen there.
