# Supplementary material for: Light regulates alternative splicing outcomes via the TOR kinase pathway
Source: Cell Rep. Author manuscript; Available in PMC 2021 Oct 26. (PMC8547716; doi:10.1016/j.celrep.2021.109676)
Supplement: Supplementary Materials [file NIHMS1745235-supplement-Supplementary_Materials.zip › 1-s2.0-S2211124721011207-mmc8.pdf]

Cell Reports

Report

Light regulates alternative splicing outcomes via the TOR kinase pathway

Graphical abstract

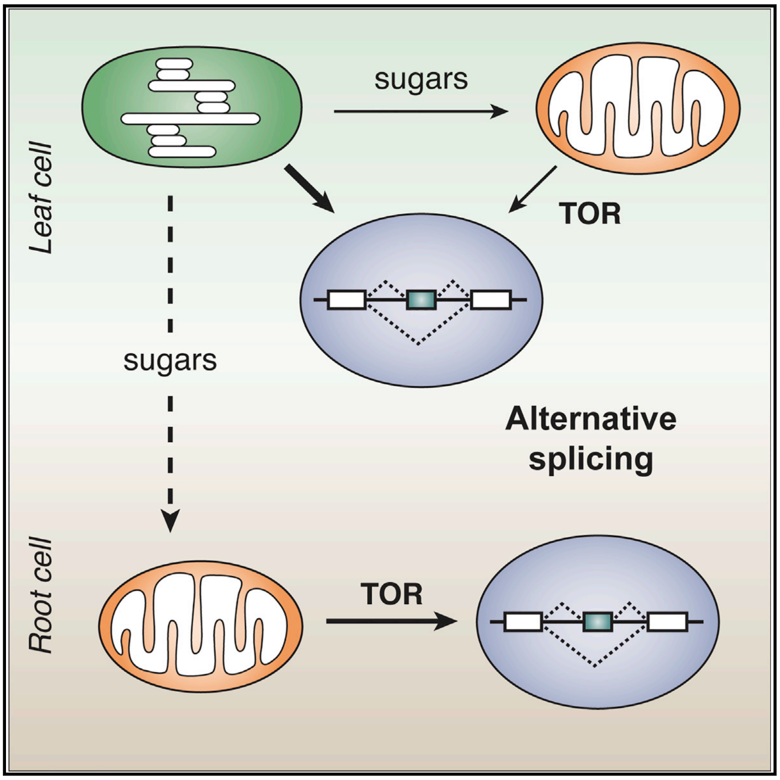

Authors

Stefan Riegler, Lucas Servi, M. Regina Scarpin, ..., Maria Kalyna, Andrea Barta, Ezequiel Petrillo

Correspondence

petry@fbmc.fcen.uba.ar

In brief

Riegler et al. reveal a central role for TOR kinase paired with retrograde signaling in alternative splicing regulation by light in roots and, to a certain extent, in leaves. Activating the TOR pathway, sugars act as mobile signals to coordinate alternative splicing responses to light throughout the whole plant.

Highlights

- Chloroplast retrograde signals control alternative splicing in *Arabidopsis thaliana*
- TOR kinase has a central role in alternative splicing control in roots
- Mitochondria mediate light/sucrose effects on alternative splicing in roots

Riegler et al., 2021, Cell Reports *36*, 109676
September 7, 2021 © 2021 The Author(s).
https://doi.org/10.1016/j.celrep.2021.109676

CellPress

Cell Reports

CellPress
OPEN ACCESS

Report

Light regulates alternative splicing outcomes via the TOR kinase pathway

Stefan Riegler,[1,9] Lucas Servi,[2,3,9] M. Regina Scarpin,[4,5] Micaela A. Godoy Herz,[2,3] María G. Kubaczka,[2,3] Peter Venhuizen,[1] Christian Meyer,[6] Jacob O. Brunkard,[4,5,7] Maria Kalyna,[1] Andrea Barta,[8] and Ezequiel Petrillo[2,3,10,*]

[1]Department of Applied Genetics and Cell Biology, BOKU – University of Natural Resources and Life Sciences, Muthgasse 18, 1190 Vienna, Austria

[2]Universidad de Buenos Aires, Facultad de Ciencias Exactas y Naturales, Departamento de Fisiología, Biología, Molecular, y Celular, Buenos Aires, Argentina

[3]CONICET-Universidad de Buenos Aires, Instituto de Fisiología, Biología Molecular y Neurociencias (IFIBYNE), C1428EHA, Buenos Aires, Argentina

[4]Department of Plant and Microbial Biology, University of California, Berkeley, Berkeley, CA 94720, USA

[5]Plant Gene Expression Center, US Department of Agriculture, Agricultural Research Service, Albany, CA 94710, USA

[6]Institut Jean-Pierre Bourgin, Institut National de la Recherche Agronomique, AgroParisTech, Centre National de la Recherche Scientifique, Université Paris-Saclay, Versailles, France

[7]Laboratory of Genetics, University of Wisconsin, Madison, Madison, WI 53706, USA

[8]Max Perutz Labs, Medical University of Vienna, Vienna Biocenter Campus, 1030 Vienna, Austria

[9]These authors contributed equally

[10]Lead contact

*Correspondence: petry@fbmc.fcen.uba.ar

https://doi.org/10.1016/j.celrep.2021.109676

SUMMARY

For plants, light is the source of energy and the most relevant regulator of growth and adaptations to the environment by inducing changes in gene expression at various levels, including alternative splicing. Light-triggered chloroplast retrograde signals control alternative splicing in *Arabidopsis thaliana*. Here, we provide evidence that light regulates the expression of a core set of splicing-related factors in roots. Alternative splicing responses in roots are not directly caused by light but are instead most likely triggered by photosynthesized sugars. The target of rapamycin (TOR) kinase plays a key role in this shoot-to-root signaling pathway. Knocking down TOR expression or pharmacologically inhibiting TOR activity disrupts the alternative splicing responses to light and exogenous sugars in roots. Consistently, splicing decisions are modulated by mitochondrial activity in roots. In conclusion, by activating the TOR pathway, sugars act as mobile signals to coordinate alternative splicing responses to light throughout the whole plant.

INTRODUCTION

Light is essential for plants: it is their source of energy and makes carbon fixation possible, allowing life on Earth as we know it. Light is also the main source of information about the ever-changing environment for plants. Since their success depends on this environmental cue, it is not surprising that plants have evolved a rich variety of mechanisms to sense light wavelength, direction, and quantity (Gyula et al., 2003; Perrella and Kaiserli, 2016). Different families of photosensory proteins are important for proper light perception. In addition, chloroplasts are gaining recognition as key components of plant photosensory networks (Chan et al., 2016; Mancini et al., 2016). The nucleus controls most aspects of organelle biogenesis and function by means of anterograde signals. Conversely, chloroplasts and mitochondria regulate nuclear gene expression with retrograde signals that modulate transcription and translation (Blanco et al., 2014; Szechyńska-Hebda and Karpiński, 2013). Previously, we reported that nuclear alternative splicing is modulated by light through a chloroplast retrograde signaling pathway (Petrillo et al., 2014) and that this process is related to an increase in transcription elongation (Godoy Herz et al., 2019). In addition, we showed that photosynthesis modulates alternative splicing responses in roots, suggesting the existence of intercellular mobile retrograde signals (Petrillo et al., 2014). The central question that now arises is how light sensed by shoot chloroplasts coordinates nuclear splicing throughout the whole plant.

Mature chloroplasts photosynthesize carbohydrates that are further metabolized in the very same leaves or loaded into the phloem to feed non-photosynthetic tissues (Brunkard, 2020). In *Arabidopsis thaliana*, the most prominent phloem-mobile sugar is sucrose (Wippel and Sauer, 2012). As is the case with light, sugars play a dual role in plant cells, serving as sources of energy and carbon as well as acting as signals (Wind et al., 2010). The roots of most terrestrial plants have translucent plastids (leukoplasts) and grow in darkness underground. However, they are able to respond to the illumination of the shoot (Sakaguchi and Watanabe, 2017). Photosynthesized sugars are essential to

This is an open access article under the CC BY license (http://creativecommons.org/licenses/by/4.0/).

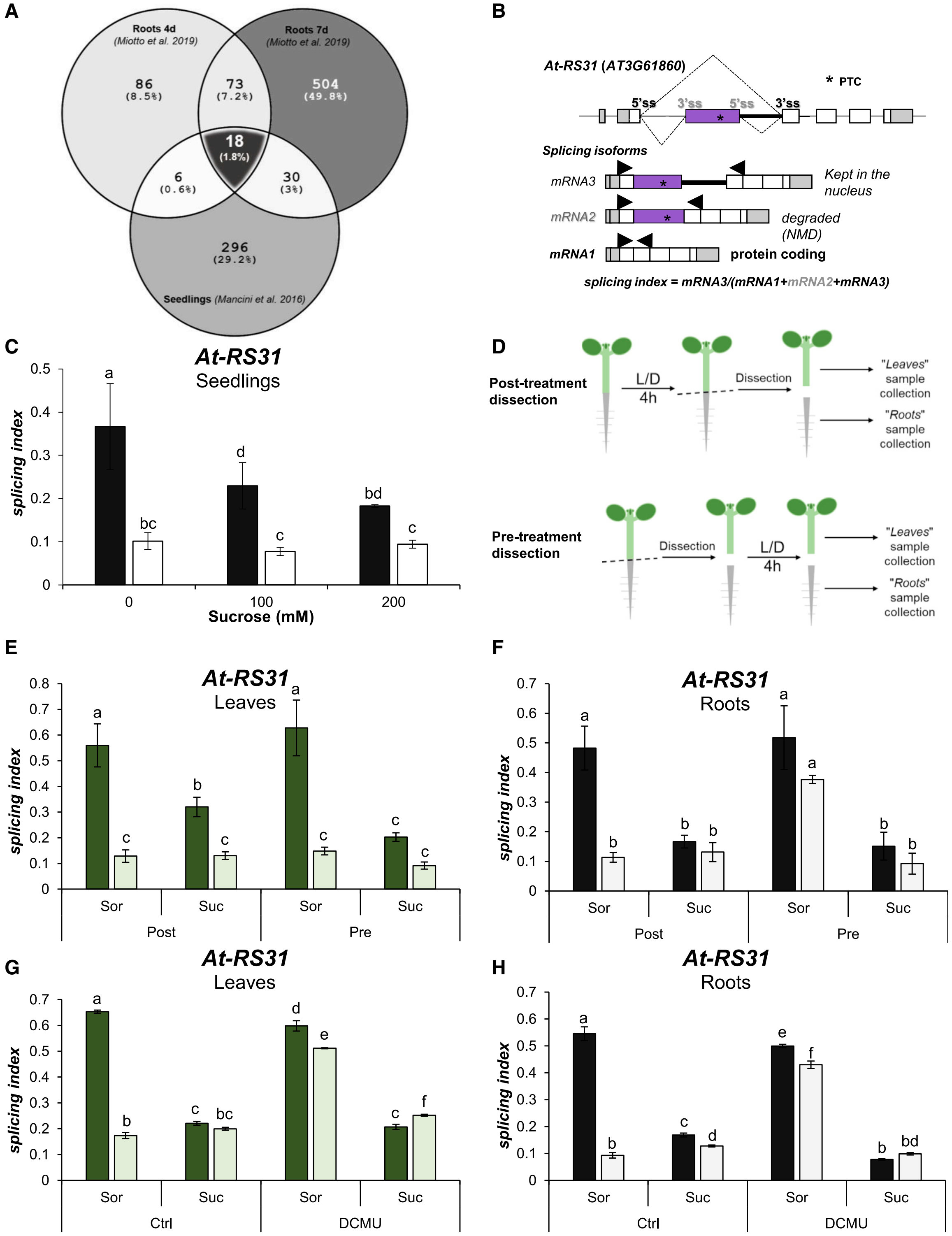

Figure 1. Sugars control alternative splicing in roots

(A) Venn diagram showing overlap between affected alternative splicing events in RNA-seq data from different experiments. Differentially alternatively spliced (DAS) genes were assessed in data from root samples, with shoots exposed to long-day conditions or darkness (Miotto et al., 2019), and data from seedling samples that received an acute light treatment at the middle of the night period (Mancini et al., 2016). Significantly affected genes are listed in Tables S1, S2, and S3.

(legend continued on next page)

induce and maintain root development (Kircher and Schopfer, 2012). Sugar levels modulate gene expression, metabolism, cell cycle, development, and adaptation to the environment (Li and Sheen, 2016; Rolland et al., 2006). Different intra- and extracellular sensors can perceive sugars. In addition to these direct pathways, where the hexokinase 1 (HXK1, AT4G29130) has a central role, sugars can be perceived by the sucrose non-fermenting related protein kinase 1 (SnRK1) and by the target of rapamycin (TOR) kinase. Sugars have been shown to repress SnRK1.1 (AT3G01090) (Baena-González et al., 2007) and to activate TOR kinase (AT1G50030) (Dobrenel et al., 2016a). Moreover, it was previously shown that sugars derived from photosynthesis activate the root meristem through TOR (Xiong et al., 2013), modulate G protein activity in a long-distance communication pathway (Tunc-Ozdemir et al., 2018), and regulate alternative splicing in etiolated seedlings (Hartmann et al., 2016). Here, we show that by activating the TOR pathway in roots, sugars act as mobile signals to coordinate alternative splicing responses to light throughout the whole plant.

RESULTS

Light regulates alternative splicing in roots

Light/dark transitions regulate the alternative splicing outcomes of several genes in *Arabidopsis thaliana* (Godoy Herz et al., 2019; Mancini et al., 2016; Petrillo et al., 2014; Shikata et al., 2014). We previously showed that light regulates alternative splicing via chloroplast-derived retrograde signals. We also found that light-induced changes in alternative splicing in roots only occur as long as communication with the photosynthetic tissue is not interrupted (Petrillo et al., 2014). Since these previous analyses were performed using a small set of genes, we expanded the study to a genome-wide level. We re-analyzed publicly available RNA-seq datasets derived from strongly contrasting conditions of light/dark incubation. We analyzed the alternative splicing responses of dark-grown roots from seedlings whose shoots were exposed to continuous darkness or long-day (LD) conditions for 4 or 7 days (Miotto et al., 2019). Using the 3-dimensional RNA sequencing (3D RNA-seq) application (Guo et al., 2020), we found 183 and 625 genes with significantly different alternative splicing patterns in roots when shoots were exposed to light (4 or 7 days in LD conditions, respectively) versus darkness (Table S1). These results suggest that light-derived signals from shoots (photosynthetic tissues) control the alternative splicing of multiple genes in dark-grown roots.

Nevertheless, it is important to note that some of the affected events may be responsive to developmental changes. Hence, to identify the events that could be directly controlled by light-triggered signals, we evaluated alternative splicing changes in response to a short light treatment in plants developed under the same growth conditions. In particular, we used RNA-seq datasets generated from seedlings that were given an acute light pulse in the middle of the night period (Mancini et al., 2016). To compare this whole-seedling experiment with the root experiments, we analyzed the former data using the 3D RNA-seq application with default settings as well. We found 350 genes with significantly different alternative splicing patterns in response to the acute light treatment (Table S2).

To identify alternative splicing events that could be directly regulated by light-triggered signals, we evaluated the overlapping events from both experiments. The root samples from 4- and 7-day-old LD- versus dark-grown seedlings share a total of 91 genes showing differential alternative splicing (DAS), and 18 of them overlap with those changing in the acute treatment (overlap significance: representation factor 19.3, $p < 2.327e{-18}$) (Figure 1; Table 1). Remarkably, among these 18 genes, we found *At-RS31* (AT3G61860), *At-SR30* (AT1G09140), and *At-U2AF65A* (AT4G36690), 3 genes previously used to characterize the chloroplast retrograde signaling pathway controlling alternative splicing in response to light (Petrillo et al., 2014). Moreover, other splicing factors are also part of this set: *At-PRP43b* (AT2G47250), *At-SR34a* (AT3G49430), and *At-RS41* (AT5G52040) (Barta et al., 2010; Wang et al., 2019). We created a tool that allows visualization of splicing isoform schematics (https://boxify.boku.ac.at/). Furthermore, Gene Ontology (GO) term overrepresentation tests of this gene set showed the highest enrichment in terms related to RNA splicing and alternative splicing (Table S3).

These findings suggest that splicing-related factors are part of a core response that fine-tunes root gene expression to cope with different light conditions perceived by the shoots. Our results further prompted us to continue using these alternatively spliced genes as reporters to gain deeper knowledge about the light-signaling mechanism that coordinates splicing responses throughout the whole plant.

Sugar mimics the effect of light on alternative splicing patterns in roots

We established that chloroplast-derived signals from leaves can reach roots and control alternative splicing outcomes in this

(B) Gene model and alternative splicing isoforms of *At-RS31*. *, PTC: premature termination codon. Arrows: primers used for splicing evaluation. The alternative usage of 5′ss and 3′ss gives rise to 3 isoforms. Usage of gray 3′ss generates mRNA3, and if the gray 5′ss is also recognized, mRNA2 (inclusion of purple exon). The coding isoform, mRNA1, is generated by the use of black 5′′ and 3′ss only.

(C) Exogenous sucrose (Suc) diminishes splicing indexes in dark-treated seedlings. Whole seedlings were treated under a light/dark protocol (Figure S1) and sucrose was added to plant growth media at 100 or 200 mM. Sorbitol (Sor) was used as osmotic control to ensure equal osmolarity (200 mM total) in all of the treatments.

(D) Post-treatment dissection was done after the light/dark treatment, immediately before sample collection. Pre-treatment dissection was done before light/dark incubation.

(E and F) Roots are not directly responsive to light but they are responsive to sugars. Incubation with sucrose (200 mM) was conducted during the light/dark treatments.

(G and H) Sugar control of alternative splicing is independent of chloroplast function. Sucrose (100 mM) and DCMU (20 μM) were added before light/dark treatments. Ethanol was used as vehicle (Ctrl). In (C) and (E)-(H), Sorbitol (Sor) was used as osmotic control at the same concentration as sucrose. Lighter bars, light; darker bars, darkness. Data represent splicing index means ± standard errors (n = 4). The same letters indicate means that are not statistically different ($p > 0.05$).

Table 1. Light controls alternative splicing of a core set of splicing-related factors

Name	Locus	Description
ADF11	AT1G01750	actin depolymerizing factor 11
At-SR30	AT1G09140	encodes a serine-arginine-rich RNA binding protein involved in regulation of splicing (including splicing of itself)
ALY3, IRP8	AT1G66260	involved in rRNA processing 8, RNA-binding (RRM/RBD/RNP motifs) family protein
AMY3	AT1G69830	α-amylase-like 3, encodes a plastid-localized α-amylase.
–	AT1G72500	inter-α-trypsin inhibitor, heavy chain-like protein
ATMYO5, ATXIF, MYOSIN 5	AT2G31900	encodes a novel myosin isoform
–	AT2G36320	A20/AN1-like zinc finger family protein
ATNCER2	AT2G38010	neutral ceramidase 2, neutral/alkaline non-lysosomal ceramidase
PRP43b	AT2G47250	RNA helicase family protein
ATMS2	AT3G03780	encodes a cytosolic methionine synthase, involved in methionine regeneration via the activated methyl cycle (or SAM cycle)
–	AT3G06530	armadillo (ARM) repeat superfamily protein, U3 small nucleolar RNA-associated protein
SR34a	AT3G49430	Serine/arginine-rich protein splicing factor 34A
At-RS31	AT3G61860	encodes an arginine/serine-rich splicing factor
–	AT4G35785	RNA-binding (RRM/RBD/RNP motifs) family protein
At-U2AF65A	AT4G36690	U2 small nuclear ribonucleoprotein auxiliary factor U2AF subunit
AtUAP56–2	AT5G11170	homolog of human UAP56 A, UAP56A, encoding an ATP-dependent RNA helicase that localizes predominantly to euchromatic regions
At-RS41	AT5G52040	Encodes an arginine/serine-rich splicing factor
LIP1	AT5G64813	the light-insensitive period1 (LIP1) gene encodes a small GTPase that influences the light input pathway of the plant circadian network

organ (Petrillo et al., 2014). A more recent report showed that sugars modulate alternative splicing in etiolated seedlings in a similar manner as light (Hartmann et al., 2016). Interestingly, splicing factors *At-SR30* and *At-SR34a* and other genes from our "core set" were used in that study to validate the results. These genes showed light- and sugar-triggered changes in their alternative splicing events in etiolated seedlings. Furthermore, sugar addition to plant growth media (Figure S1A) diminishes the splicing index of *At-RS31* when analyzing whole seedlings (Figures 1B and 1C), resembling the action of light on this splicing event (Petrillo et al., 2014). Hence, we hypothesized that in photosynthetically active seedlings, sugars produced in photosynthetic cells are the main drivers of light-regulated alternative splicing in non-photosynthetic cells. To investigate this, we analyzed the splicing responses of leaves and roots separately. Importantly, when light/dark treatments are performed on whole plants (Figure 1D), light regulates *At-RS31* alternative splicing in leaves (Figure 1E) and roots (Figure 1F). When plants are dissected before the light/dark treatment (Figure 1D), however, roots disconnected from photosynthetic tissues lose the capacity to change the alternative splicing of *At-RS31* in response to light (Figure 1F). In this treatment scheme, sucrose addition completely recapitulates light effects in roots, detached or not (Figure 1F), with milder influence in leaves (Figure 1E). These results demonstrate that sucrose modulates nuclear alternative splicing of *At-RS31,* especially in root cells. Since the difference between the effect of sucrose in leaves and roots may be due to an inefficient sugar uptake by leaves from the agar-growth media, we submerged the plants in liquid growth medium and applied vacuum infiltration. Using 100 mM sucrose in these conditions mimics light effects on the alternative splicing patterns of *At-RS31*, *At-U2AF65A*, and *At-SR30* in leaves as in roots (Figures 1G, 1H, and S1B). Furthermore, blocking the photosynthetic electron transport with DCMU (3-(3,4-dichlorophenyl)-1,1-dimethylurea; Karpinski et al., 1997) does not disrupt sucrose effects on these alternative splicing events (Figures 1G,1H, and S1B), indicating that sugar effects are independent of the chloroplasts. Even when vacuum infiltration was used, root cells are more sensitive to sucrose for some of the analyzed alternative splicing events (Figures 1E–1H and S1B). In addition, when chloroplast function is disrupted, leaves show similar responses to light and sugars as roots (Figures 1G and 1H). These results indicate that sugars derived from photosynthesis, such as sucrose, can control roots' alternative splicing responses in the same fashion as light.

TOR kinase controls alternative splicing responses to light and sugars in roots

Light drives photosynthesis of carbohydrates that are metabolized to various sugars. These compounds have been reported to modulate the activity of different plant kinases, including HXK1, SnRK1, and TOR (Li and Sheen, 2016), that in turn, control gene expression. Since we previously studied and ruled out HXK1 and SnRK1 as sensors for this light signaling pathway (Petrillo et al., 2014), we decided to assess the involvement of the TOR pathway.

First, we took a global transcriptomic approach. Building on the experimental design by Xiong and colleagues, we grew

A. thaliana seedlings to quiescence (Xiong et al., 2013) and then treated them for 2 h with a physiological concentration of glucose (15 mM) to activate TOR, or with glucose and Torin2 (5 μM) to attenuate TOR activity (Scarpin et al., 2020). We then performed RNA-seq and analyzed the data with the 3D RNA-seq application to assess differences in gene expression and alternative splicing. We found 1,054 differentially expressed genes using stringent cutoff parameters (|fold-change| > 2, p < 0.01; Table S4), which massively overlapped with previous transcriptome-wide analyses of TOR signaling in *A. thaliana*, indicating that our experimental approach affected TOR activity as expected. Strikingly, we identified 160 genes with differential transcript utilization (DTU; Table S5). Significant DTU of 19 of these genes was also detected in at least one of the light-sensitive RNA-seq experiments analyzed above. These genes included *RIK* (AT3G29390), an RNA-binding K homology (KH) protein that forms a complex with developmental regulators AS1 and AS2; *APT5* (AT5G11160), an adenine phosphoribosyltransferase acting in AMP salvage; and *RPP2A* (AT4G19500), a TIR-NBS-LRR (TNL) receptor that is required for RPP2-mediated resistance to *Peronospora parasitica* isolate Cala2. We validated the DTU results by conducting the seedling treatment assay another three times, independently, and analyzing the splicing of *At-RS31* and the above-mentioned genes by RT-PCR (Figure S2A).

Seeing as TOR kinase activity modulates alternative splicing, we wondered whether members of the core light response of the roots (Table 1) are affected by the TOR pathway. Light/dark-treated TOR RNAi transgenic plants (Deprost et al., 2007) show a small reduction in *At-RS31* alternative splicing responses in leaves (Figure 2A), evidencing a minor contribution of TOR kinase in this organ. However, this TOR knockdown line shows a complete abolishment of the light effects on the alternative splicing of *At-RS31* in roots (Figure 2B). *At-U2AF65A* and *At-SR30* alternative splicing events also show disrupted regulation in the roots of this TOR-RNAi line (Figure S2B). These results suggest a role for TOR kinase in alternative splicing regulation by light in root cells. Since plants with decreased levels of TOR kinase develop considerably shorter roots (Deprost et al., 2007), we decided to use a specific inhibitor to exclude an indirect growth effect. By using 20 μM AZD-8055, an ATP competitive TOR kinase inhibitor (Montané and Menand, 2013), light-induced changes on *At-RS31* alternative splicing in leaves are reduced (Figure 2C). More interesting is that TOR inhibition by AZD-8055 completely abolishes *At-RS31* alternative splicing changes triggered by light in roots (Figure 2D). The results obtained using the TOR RNAi line and different TOR inhibitors support a key role of TOR kinase activity in the light-signaling pathway that controls alternative splicing in roots.

Moreover, we analyzed the alternative splicing patterns of the core light response splicing- related gene set using 2 AZD-8055 concentrations, a 20-μM dose (as in previous experiment; Figure 2C) and a lower dose, 2 μM, which is often used when applying longer treatments (Montané and Menand, 2013). AZD-8055 treatment during the light/dark incubation (~4 h) disrupts light and sucrose effects on the alternative splicing modulation of *At-RS31* and *At-U2AF65A* (Figure S3) in both leaves and roots, with a stronger effect in the latter. Similar but milder effects are shown by *At-SR30* and *At-SR34a* alternative splicing patterns (Figure S3). The higher AZD-8055 dose (20 μM) was most effective in all cases (Figure S3). Importantly, this dose did not cause major changes in a pair of splicing factors, *At-SCL30* (AT3G55460) and *At-SCL33* (AT1G55310), that are also light and sugar responsive (Figure S3). This indicates that when applied for a short time, this high AZD-8055 dose does not globally disrupt alternative splicing responses to light and/or sugars.

Since roots are not responsive to light per se (Figure 1F) and TOR kinase is a sensor that integrates stress-, nutrient-, and energy-related signals (Schepetilnikov and Ryabova, 2018), we hypothesized that this signaling pathway controls alternative splicing in roots in response to sugars. We used isolated (detached) roots and treated them with sucrose and AZD-8055 to test this hypothesis. Figure S4 supports this notion, since AZD-8055 treatment disrupts sucrose-induced changes on *At-RS31*, *At-U2AF65A*, and *At-SR30* alternative splicing events in isolated roots.

These results confirm that TOR kinase is involved in the alternative splicing control executed by light and sugars in plants, being of key relevance in roots.

Chloroplasts and mitochondria regulate TOR pathway activity

Since the TOR signaling pathway modulates alternative splicing, we next asked whether chloroplast signals upon light irradiation could control TOR kinase activity. We used phosphorylation of Ser240 of the ribosomal protein subunit 6 (RPS6) as a proxy for TOR pathway activity (Dobrenel et al., 2016b). Western blots show, as expected, that light activates the TOR signaling pathway when leaves (Figure 2E) and roots (Figure 2F) are transferred from extended darkness to light, as evidenced by the increased phosphorylation of RPS6-S240. AZD-8055 efficiently inhibits light-activated RPS6-S240 phosphorylation (Figures 2E and 2F, upper panels). *At-RS31* alternative splicing responses correlate well with the activity of the TOR pathway. In fact, the higher the phosphorylation level of RPS6-S240, the lower the splicing index (Figures 2E and 2F). In addition, similar results were obtained with *At-U2AF65A* and *At-SR30* (Figure S5), indicating a common regulation. Importantly, the induction of RPS6-S240 phosphorylation by sucrose and its inhibition by AZD-8055, in leaves as well as in roots, rule out differences in the uptake and/or efficacy of these compounds between tissues. Hence, TOR activity is modulated by light and sugars in our experimental system, and TOR activity closely correlates with alternative splicing responses to these stimuli.

Since the signaling pathway that controls *At-RS31* alternative splicing involves the chloroplast (Figures 1G and 1H; Petrillo et al., 2014), we asked whether blocking the photosynthetic electron transport would also abolish TOR activation triggered by light or whether TOR kinase activity is modulated by the chloroplast. In line with Xiong et al. (2013), blocking the chloroplast electron transport chain with DCMU inhibits RPS6 phosphorylation in response to light in leaves (Figure S6A). These results indicate that chloroplasts control TOR kinase activity in leaves, but how this signaling pathway is activated in roots to control alternative splicing remains to be determined. Xiong et al. (2013) have shown that the activation of the TOR pathway in roots

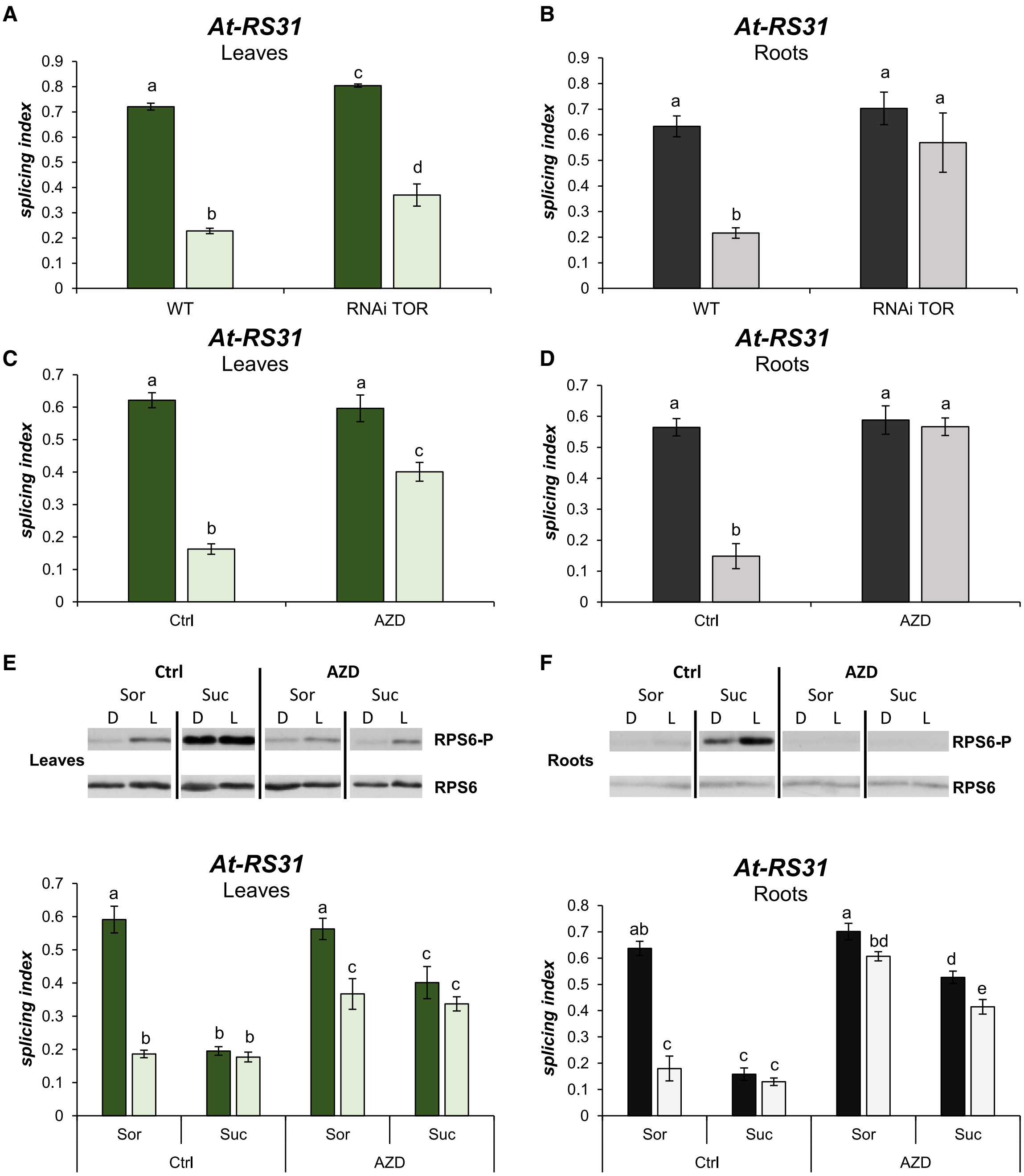

Figure 2. TOR kinase modulates alternative splicing responses to light and sugars in roots

(A and B) Knock down of TOR kinase expression abolishes alternative splicing changes induced by light in roots. RNAi TOR, transgenic line expressing an RNAi against TOR kinase mRNA. WT, wild-type seedlings. Splicing indexes for *At-RS31* in response to light/dark are shown for leaves (A) and roots (B).

(C and D) TOR kinase activity is necessary for light-mediated alternative splicing response in roots. TOR kinase inhibitor, AZD-8055 (AZD), was used during the light/dark treatment. Splicing indexes for *At-RS31* in response to light/dark are shown for leaves (C) and roots (D). Dimethyl sulfoxide was used as a vehicle (Ctrl).

(E and F) The TOR kinase pathway is activated by light and sugars. Upper panels, western blot images of RPS6 phosphorylation (RPS6-P) and total RPS6 levels in light/dark treated seedlings with exogenous sucrose (Suc, 100 mM) in leaves (E) and roots (F). Bottom graphs, splicing indexes in leaves (E) and roots (F) corresponding to the samples in the upper panels. Sorbitol (Sor, 100 mM) was used as osmotic control. (A-F) Lighter bars, light; darker bars, darkness. Graphs show means ± standard errors (n = 4). The same letters indicate means that are not statistically different ($p > 0.05$).

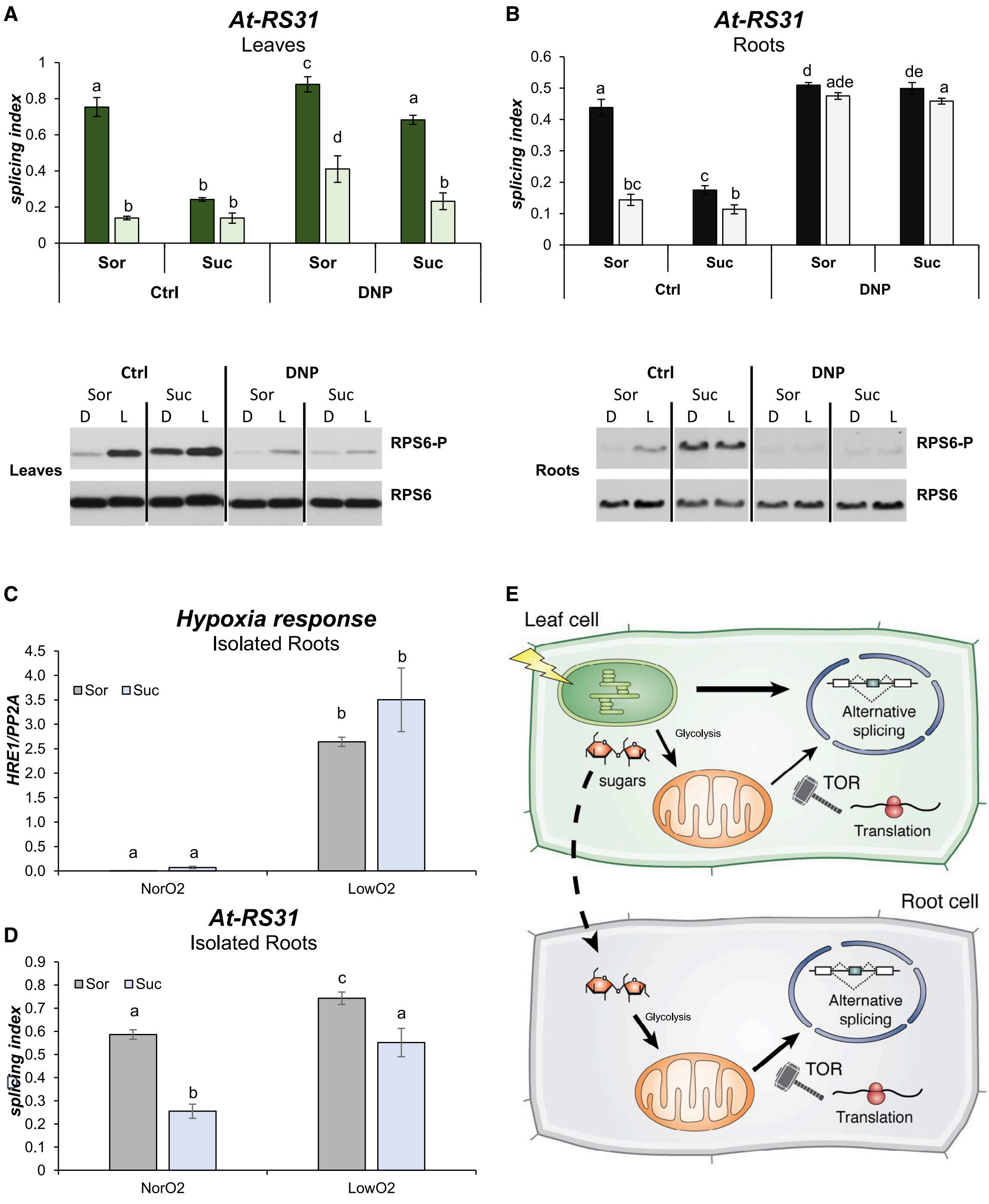

Figure 3. Mitochondria control alternative splicing in roots through TOR kinase activity

(A and B) Disrupting proton gradients abolishes light/dark splicing changes and TOR kinase activity modulation in roots. The ionophore 2,4-dinitrophenol (DNP) was used in a 20-μM concentration to incubate plants under the light/dark protocol. Suc, sucrose 100 mM. Sor, sorbitol 100 mM as osmotic control. Splicing indexes of *At-RS31* are shown for leaves (A) and roots (B) in the top panels. Lighter bars, light; darker bars, darkness. Data represent splicing index means ± standard error (n = 4). The same letters indicate means that are not statistically different ($p > 0.05$). Bottom panels are western blot images of RPS6 phosphorylation (RPS6-P) and total RPS6 levels in leaves (A) and in roots (B).

(C and D) Low oxygen levels cause reduced alternative splicing responses. Plants were grown in constant light for 2 weeks, transferred to darkness for 48 h. Seedlings were dissected and isolated roots were treated with 50 mM sucrose or sorbitol in the presence of low oxygen (LowO2) or air (NorO2). Data represent splicing index means ± standard errors (n = 4). The same letters indicate means that are not statistically different ($p > 0.05$).

(C) *HRE1* (hypoxia-inducible ethylene response factor 1) expression.

(legend continued on next page)

occurs via sugars derived from photosynthesis that, after glycolysis, feed the mitochondria (. Since we demonstrated that alternative splicing regulation by light in roots is driven by sugars and not directly by light, we reasoned that a pathway, similar to the one described by Xiong et al. (2013), could act to control alternative splicing in roots. Following this line of thought, uncoupling the energy generation from the electron transport in the organelles using an ionophore (2,4-dinitrophenol [DNP]) should disrupt the alternative splicing changes induced by light and sucrose. This uncoupler has only a mild effect on light-mediated *At-RS31* alternative splicing changes in leaves. Interestingly, DNP clearly reduces the effects of sucrose, partially restoring light/dark responses, not only in terms of alternative splicing regulation but also regarding TOR kinase activity (Figure 3A; Xiong et al., 2013). Roots, however, show a more dramatic response: DNP abolishes the changes in *At-RS31* alternative splicing induced by light and/or sucrose as well as the activation of the TOR pathway in roots (Figure 3B). Similar results were obtained for the alternative splicing events of *At-U2AF65A* and *At-SR30* (Figure S6B). Furthermore, for all of these events, DNP treatment increases the light splicing indexes in leaves, mirroring the effects of AZD-8055 and the TOR RNAi knockdown line in roots. These results indicate that mitochondria have a key role in the light/sugar signaling pathway that controls alternative splicing in roots through the activation of TOR kinase.

Since a pharmacological treatment could be causing indirect effects, we used an inhibitor-independent approach to validate the role of mitochondria and TOR kinase on root alternative splicing regulation. We generated a low oxygen atmosphere replacing ambient air with nitrogen-enriched air in a closed environment. A remarkable increase in the expression of a hypoxia-responsive gene, *HRE1* (Licausi et al., 2010), confirmed the low levels of oxygen (Figure 3C). Under these conditions, which imply low levels of mitochondrial function, isolated roots show reduced *At-RS31* alternative splicing changes in response to sucrose (Figure 3D). These results indicate that the alternative splicing regulation of different genes in root cells is linked to the activity of the mitochondria and the concomitant activation of the TOR kinase pathway.

DISCUSSION

Our study reveals a central role for TOR kinase in alternative splicing regulation by light in non-photosynthetic tissues (roots) and, to a certain extent, also in photosynthetic tissues (leaves). TOR activation in roots is triggered by sugars that feed glycolysis and then activate mitochondria (Xiong et al., 2013). Disrupting mitochondrial activity abolishes the light/sucrose-triggered changes on alternative splicing in roots and causes a striking inhibition of RPS6-S240 phosphorylation (Figures 3 and S6B). We now have a more complete picture about how alternative splicing is regulated by light throughout the whole plant. Light is initially sensed by the chloroplast and activates photosynthesis. Synthesized sugars are loaded into the phloem and travel to non-photosynthetic root tissues. There, imported sugars are metabolized through glycolysis, yielding pyruvate that enters the mitochondria. Pyruvate fuels oxidative phosphorylation in the mitochondria that activates TOR and, in turn, modulates alternative splicing outcomes (Figure 3E). In leaves, even though a similar mechanism seems to be active, there are other retrograde signals derived from the photosynthetic electron transport (DCMU sensitive) that can modulate nuclear splicing decisions. These other mechanisms appear to be independent of TOR kinase, as AZD-8055 and DNP did not completely abolish light-triggered alternative splicing changes in the photosynthetic tissues. However, the phosphorylation of RPS6-S240, which correlates well with splicing changes, may suggest an involvement of the TOR pathway. To what extent TOR kinase contributes to light-dependent alternative splicing regulation in leaves, as well as how that contribution is achieved exactly, are questions that need further investigation. Interestingly, mammalian TOR regulates U2 auxiliary factor 1 (U2AF1) splicing, showing that this highly conserved pathway could be acting as a splicing modulator in different organisms (Chang et al., 2019). Since TOR kinase is a major regulator of mRNA translation in all eukaryotic cells (Chen et al., 2018; Laplante and Sabatini, 2012; Scarpin et al., 2020), and light/dark transitions are also known to modulate translation in plants (Liu et al., 2012), we speculate that light and sugars could activate the translation of a factor that, in turn, modulates nuclear splicing decisions (Figure 3E). Alternatively, TOR could post-translationally modulate the activity of factors involved in transcription or splicing regulation; many RNA-binding proteins involved in processing and splicing are significantly differentially phosphorylated in response to TOR activity (Scarpin et al., 2020). Different signaling mechanisms could converge in the activation of translation in the cytosol. Interestingly, it was shown that TOR transmits light signals that enhance translation in de-etiolating seedlings (Chen et al., 2018). The participation of a newly translated protein (e.g., a splicing regulator) that can move to the nucleus could be the missing piece in the communication between different organelles, a crucial and elusive component of retrograde signaling mechanisms (Figure 3E). However, other mechanisms connecting TOR activity with nuclear decisions in response to sugar were recently reported (Fu et al., 2021). Further research is needed to shed more light on the underlying processes linking TOR kinase activity with splicing decisions and its outcomes.

STAR★METHODS

Detailed methods are provided in the online version of this paper and include the following:

- KEY RESOURCES TABLE
- RESOURCE AVAILABILITY
  - Lead contact

(D) *At-RS31* alternative splicing index.
(E) Working model: different retrograde signals regulate alternative splicing through inter-organellar communication. Photosynthesis in the chloroplasts generates different signals that modulate alternative splicing in the nucleus of photosynthetic leaf cells. Among these signals, sucrose is also transported to the roots and feeds the mitochondria after glycolysis and activates TOR, thus changing alternative splicing in this heterotrophic tissue. Art by Dr. Luciana Giono.

- Materials availability
- Data and code availability
- EXPERIMENTAL MODEL AND SUBJECT DETAILS
  - Plant material and growth conditions
- METHOD DETAILS
  - Pharmacological, sugar and low oxygen treatments
  - RT- PCRs for alternative splicing assessment
  - RT-qPCR expression analysis
  - Western blot
- QUANTIFICATION AND STATISTICAL ANALYSIS
  - Statistics
  - 3D RNA-seq app analyses
  - Venn Diagrams
  - Gene Ontology classification
- ADDITIONAL RESOURCES
  - Alternative splicing isoforms web tool

SUPPLEMENTAL INFORMATION

Supplemental information can be found online at https://doi.org/10.1016/j.celrep.2021.109676.

ACKNOWLEDGMENTS

We thank A.R. Kornblihtt, R. Hausler, J. Sheen, A. Köhler, P. Duque, Y. Marquez, and C. Simpson for materials, discussions, and advice. We especially acknowledge the input, feedback, and lessons from "Big John," J.W.S. Brown. We also thank the "307," Kornblihtt, Srebrow, Barta, and Kalyna groups for the great working atmospheres; A.E. Cambindo Botto, D. Reifer, Viole, and Mbututu for extensive and exciting discussions; the IFIBYNE technicians for their support; and L. Giono for her awesome art. This work was supported by the Austrian Science Fund FWF (P26333, to M.K.; DK W1207 and SFBF43-P10, to A.B.), the US National Institutes of Health (NIH; DP5-OD023072, to J.O.B.), and the Agencia Nacional de Promoción de Ciencia y Tecnología of Argentina to E.P. (PICT 2016-4366, PICT 2017-1343, PICT 2019-01690). E.P. was an EMBO long-term fellow (ALTF_1337-2012) and a Marie Curie postdoctoral fellow P330888 (ASRNAbidoPhys) and is now a career investigator from the Consejo Nacional de Investigaciones Científicas y Técnicas of Argentina.

AUTHOR CONTRIBUTIONS

E.P. designed the research and, together with S.R. and L.S., performed most of the experiments with the assistance of M.G.K. and M.A.G.H. for specific assays. M.R.S. and J.O.B. designed and performed the Torin2 RNA-seq experiment and validation. P.V. contributed computational tools and support for bioinformatic analyses. C.M. provided input and resources for the TOR activity analyses. A.B., M.K., and E.P. analyzed the data and wrote the paper, with feedback from all of the authors.

DECLARATION OF INTERESTS

The authors declare no competing interests.

Received: September 25, 2017
Revised: May 18, 2021
Accepted: August 16, 2021
Published: September 7, 2021

REFERENCES

Baena-González, E., Rolland, F., Thevelein, J.M., and Sheen, J. (2007). A central integrator of transcription networks in plant stress and energy signalling. Nature *448*, 938–942.

Barta, A., Kalyna, M., and Reddy, A.S.N. (2010). Implementing a rational and consistent nomenclature for serine/arginine-rich protein splicing factors (SR proteins) in plants. Plant Cell *22*, 2926–2929.

Benjamini, Y., and Yekutieli, D. (2001). The control of the False Discovery Rate in multiple testing under dependency. Ann. Stat. *29*, 1165–1188.

Blanco, N.E., Guinea-Díaz, M., Whelan, J., and Strand, Å. (2014). Interaction between plastid and mitochondrial retrograde signalling pathways during changes to plastid redox status. Philos. Trans. R. Soc. Lond. B Biol. Sci. *369*, 20130231.

Brunkard, J.O. (2020). Exaptive Evolution of Target of Rapamycin Signaling in Multicellular Eukaryotes. Dev. Cell *54*, 142–155.

Bullard, J.H., Purdom, E., Hansen, K.D., and Dudoit, S. (2010). Evaluation of statistical methods for normalization and differential expression in mRNA-Seq experiments. BMC Bioinformatics *11*, 94.

Calixto, C.P.G., Guo, W., James, A.B., Tzioutziou, N.A., Entizne, J.C., Panter, P.E., Knight, H., Nimmo, H.G., Zhang, R., and Brown, J.W.S. (2018). Rapid and Dynamic Alternative Splicing Impacts the Arabidopsis Cold Response Transcriptome. Plant Cell *30*, 1424–1444.

Chan, K.X., Phua, S.Y., Crisp, P., McQuinn, R., and Pogson, B.J. (2016). Learning the Languages of the Chloroplast: Retrograde Signaling and Beyond. Annu. Rev. Plant Biol. *67*, 25–53.

Chang, J.W., Yeh, H.S., Park, M., Erber, L., Sun, J., Cheng, S., Bui, A.M., Fahmi, N.A., Nasti, R., Kuang, R., et al. (2019). mTOR-regulated U2af1 tandem exon splicing specifies transcriptome features for translational control. Nucleic Acids Res. *47*, 10373–10387.

Chen, G.-H., Liu, M.-J., Xiong, Y., Sheen, J., and Wu, S.-H. (2018). TOR and RPS6 transmit light signals to enhance protein translation in deetiolating *Arabidopsis* seedlings. Proc. Natl. Acad. Sci. USA *115*, 12823–12828.

Deprost, D., Yao, L., Sormani, R., Moreau, M., Leterreux, G., Nicolaï, M., Bedu, M., Robaglia, C., and Meyer, C. (2007). The Arabidopsis TOR kinase links plant growth, yield, stress resistance and mRNA translation. EMBO Rep. *8*, 864–870.

Dobrenel, T., Caldana, C., Hanson, J., Robaglia, C., Vincentz, M., Veit, B., and Meyer, C. (2016a). TOR Signaling and Nutrient Sensing. Annu. Rev. Plant Biol. *67*, 261–285.

Dobrenel, T., Mancera-Martínez, E., Forzani, C., Azzopardi, M., Davanture, M., Moreau, M., Schepetilnikov, M., Chicher, J., Langella, O., Zivy, M., et al. (2016b). The Arabidopsis TOR Kinase Specifically Regulates the Expression of Nuclear Genes Coding for Plastidic Ribosomal Proteins and the Phosphorylation of the Cytosolic Ribosomal Protein S6. Front. Plant Sci. *7*, 1611.

Fu, L., Liu, Y., Qin, G., Wu, P., Zi, H., Xu, Z., Zhao, X., Wang, Y., Li, Y., Yang, S., et al. (2021). The TOR-EIN2 axis mediates nuclear signalling to modulate plant growth. Nature *591*, 288–292.

Godoy Herz, M.A., Kubaczka, M.G., Brzyżek, G., Servi, L., Krzyszton, M., Simpson, C., Brown, J., Swiezewski, S., Petrillo, E., and Kornblihtt, A.R. (2019). Light Regulates Plant Alternative Splicing through the Control of Transcriptional Elongation. Mol. Cell *73*, 1066–1074.e3.

Guo, W., Tzioutziou, N.A., Stephen, G., Milne, I., Calixto, C.P.G., Waugh, R., Brown, J.W.S., and Zhang, R. (2020). 3D RNA-seq: a powerful and flexible tool for rapid and accurate differential expression and alternative splicing analysis of RNA-seq data for biologists. RNA Biol. https://doi.org/10.1080/15476286.2020.1858253.

Gyula, P., Schäfer, E., and Nagy, F. (2003). Light perception and signalling in higher plants. Curr. Opin. Plant Biol. *6*, 446–452.

Hartmann, L., Drewe-Boß, P., Wießner, T., Wagner, G., Geue, S., Lee, H.-C., Obermüller, D.M., Kahles, A., Behr, J., Sinz, F.H., et al. (2016). Alternative Splicing Substantially Diversifies the Transcriptome during Early Photomorphogenesis and Correlates with the Energy Availability in Arabidopsis. Plant Cell *28*, 2715–2734.

Karpinski, S., Escobar, C., Karpinska, B., Creissen, G., and Mullineaux, P.M. (1997). Photosynthetic electron transport regulates the expression of cytosolic ascorbate peroxidase genes in Arabidopsis during excess light stress. Plant Cell *9*, 627–640.

Kircher, S., and Schopfer, P. (2012). Photosynthetic sucrose acts as cotyledon-derived long-distance signal to control root growth during early seedling development in Arabidopsis. Proc. Natl. Acad. Sci USA *109*, 11217–21109.

Laplante, M., and Sabatini, D.M. (2012). mTOR signaling in growth control and disease. Cell *149*, 274–293.

Law, C.W., Chen, Y., Shi, W., and Smyth, G.K. (2014). voom: precision weights unlock linear model analysis tools for RNA-seq read counts. Genome Biol. *15*, R29.

Li, L., and Sheen, J. (2016). Dynamic and diverse sugar signaling. Curr. Opin. Plant Biol. *33*, 116–125.

Licausi, F., van Dongen, J.T., Giuntoli, B., Novi, G., Santaniello, A., Geigenberger, P., and Perata, P. (2010). HRE1 and HRE2, two hypoxia-inducible ethylene response factors, affect anaerobic responses in Arabidopsis thaliana. Plant J. *62*, 302–315.

Liu, M.-J., Wu, S.-H., Chen, H.-M., and Wu, S.-H. (2012). Widespread translational control contributes to the regulation of Arabidopsis photomorphogenesis. Mol. Syst. Biol. *8*, 566.

Mancini, E., Sanchez, S.E., Romanowski, A., Schlaen, R.G., Sanchez-Lamas, M., Cerdán, P.D., and Yanovsky, M.J. (2016). Acute Effects of Light on Alternative Splicing in Light-Grown Plants. Photochem. Photobiol. *92*, 126–133.

Mi, H., Muruganujan, A., Huang, X., Ebert, D., Mills, C., Guo, X., and Thomas, P.D. (2019). Protocol Update for large-scale genome and gene function analysis with the PANTHER classification system (v.14.0). Nat. Protoc. *14*, 703–721.

Miotto, Y.E., Tesser da Costa, C., de Oliveira, B.H., Guzman, F., Margis, R., de Almeida, R.M.C., Offringa, R., and Dos Santos Maraschin, F. (2019). Identification of root transcriptional responses to shoot illumination in Arabidopsis thaliana. Plant Mol. Biol. *101*, 487–498.

Montané, M.H., and Menand, B. (2013). ATP-competitive mTOR kinase inhibitors delay plant growth by triggering early differentiation of meristematic cells but no developmental patterning change. J. Exp. Bot. *64*, 4361–4374.

Patro, R., Duggal, G., Love, M.I., Irizarry, R.A., and Kingsford, C. (2017). Salmon provides fast and bias-aware quantification of transcript expression. Nat. Methods *14*, 417–419.

Perrella, G., and Kaiserli, E. (2016). Light behind the curtain: photoregulation of nuclear architecture and chromatin dynamics in plants. New Phytol. *212*, 908–919.

Petrillo, E., Godoy Herz, M.A., Fuchs, A., Reifer, D., Fuller, J., Yanovsky, M.J., Simpson, C., Brown, J.W.S., Barta, A., Kalyna, M., et al. (2014). A chloroplast retrograde signal regulates nuclear alternative splicing. Science *344*, 427–430.

Ritchie, M.E., Phipson, B., Wu, D., Hu, Y., Law, C.W., Shi, W., and Smyth, G.K. (2015). limma powers differential expression analyses for RNA-sequencing and microarray studies. Nucleic Acids Res. *43*, e47.

Rolland, F., Baena-Gonzalez, E., and Sheen, J. (2006). Sugar sensing and signaling in plants: conserved and novel mechanisms. Annu. Rev. Plant Biol. *57*, 675–709.

Sakaguchi, J., and Watanabe, Y. (2017). Light perception in aerial tissues enhances DWF4 accumulation in root tips and induces root growth. Sci. Rep. *7*, 1808.

Scarpin, M.R., Leiboff, S., and Brunkard, J.O. (2020). Parallel global profiling of plant TOR dynamics reveals a conserved role for LARP1 in translation. eLife *9*, 1–34.

Schepetilnikov, M., and Ryabova, L.A. (2018). Recent discoveries on the role of tor (target of rapamycin) signaling in translation in plants. Plant Physiol. *176*, 1095–1105.

Shikata, H., Hanada, K., Ushijima, T., Nakashima, M., Suzuki, Y., and Matsushita, T. (2014). Phytochrome controls alternative splicing to mediate light responses in Arabidopsis. Proc. Natl. Acad. Sci. USA *111*, 18781–18786.

Soneson, C., Love, M.I., and Robinson, M.D. (2015). Differential analyses for RNA-seq: transcript-level estimates improve gene-level inferences. F1000Res. *4*, 1521.

Szechyńska-Hebda, M., and Karpiński, S. (2013). Light intensity-dependent retrograde signalling in higher plants. J. Plant Physiol. *170*, 1501–1516.

Tunc-Ozdemir, M., Liao, K.-L., Ross-Elliott, T.J., Elston, T.C., and Jones, A.M. (2018). Long-distance communication in Arabidopsis involving a self-activating G protein. Plant Direct *2*, e00037.

Wang, J., Chen, S., Jiang, N., Li, N., Wang, X., Li, Z., Li, X., Liu, H., Li, L., Yang, Y., et al. (2019). Spliceosome disassembly factors ILP1 and NTR1 promote miRNA biogenesis in Arabidopsis thaliana. Nucleic Acids Res. *47*, 7886–7900.

Wind, J., Smeekens, S., and Hanson, J. (2010). Sucrose: metabolite and signaling molecule. Phytochemistry *71*, 1610–1614.

Wippel, K., and Sauer, N. (2012). Arabidopsis SUC1 loads the phloem in suc2 mutants when expressed from the SUC2 promoter. J. Exp. Bot. *63*, 669–679.

Xiong, Y., McCormack, M., Li, L., Hall, Q., Xiang, C., and Sheen, J. (2013). Glucose-TOR signalling reprograms the transcriptome and activates meristems. Nature *496*, 181–186.

Zhang, R., Calixto, C.P.G., Marquez, Y., Venhuizen, P., Tzioutziou, N.A., Guo, W., Spensley, M., Entizne, J.C., Lewandowska, D., Ten Have, S., et al. (2017). A high quality Arabidopsis transcriptome for accurate transcript-level analysis of alternative splicing. Nucleic Acids Res. *45*, 5061–5073.

CellPress
OPEN ACCESS

STAR★METHODS

KEY RESOURCES TABLE

REAGENT or RESOURCE	SOURCE	IDENTIFIER
Antibodies		
α-RPS6-phospho-S240 (RPS6-P)	Dobrenel et al., 2016b	N/A
α-RPS6-Total	Christian Meyer, Dobrenel et al., 2016b	N/A
Goat anti-rabbit Immunoglobulin G (IgG) Horseradish Peroxidase (HRP) Conjugate	Cell Signaling Technology	Cat# 7074; RRID:AB_2099233
Chemicals, peptides, and recombinant proteins		
DCMU, 3-(3,4-dichlophenyl)-1,1-dimethylurea	Sigma-Aldrich	Cat# D2425; CAS# 330-54-1; PubChem Substance ID 57654085
DNP, 2,4-dinitrophenol	Sigma-Aldrich	Cat# D198501; CAS# 51-28-5; PubChem Substance ID 24893583
AZD-8055	Chemdea LLC	Cat# CD0348
Torin2	Cayman Chemical	Cat# 14185; CAS# 1223001-51-1
Deposited data		
Torin2 RNA-seq data	NCBI SRA, Scarpin et al., 2020	Project PRJNA639161
Root RNA-seq data 4- and 7-days old seedlings	Gene Expression Omnibus (GEO) database, Miotto et al., 2019	Accession number GEO: GSE132249
Acute light treatment	Gene Expression Omnibus (GEO) database, Mancini et al., 2016	Accession number GEO: GSE68560
Western blots, RT-PCR and RT-qPCR data	Mendeley	https://doi.org/10.17632/xt8sznfw4s.1
Experimental models: organisms/strains		
Arabidopsis thaliana Col 0	Dr. M. Yanovsky (FIL, Buenos Aires, Argentina)	N/A
Tor RNAi line, RNAi line of TOR (TARGET OF RAPAMYCIN) 35-7	Dr. Christian Meyer, Deprost et al., 2007	N/A
Oligonucleotides		
Primers for RT-PCR splicing analysis, see Table S6	This paper	N/A
Primers for RT-qPCR, see Table S6	This paper	N/A
Software and algorithms		
Infostat	https://www.infostat.com.ar/	N/A
3D RNA-seq App	https://github.com/wyguo/ThreeDRNAseq; https://3drnaseq.hutton.ac.uk/app_direct/3DRNAseq/; Guo et al., 2020.	N/A
Galaxy - Salmon	https://usegalaxy.org/; Salmon tool; Patro et al., 2017.	N/A
Boxify – Drawing isoforms and checking RT-PCR product sizes	https://boxify.boku.ac.at/; this paper.	N/A

RESOURCE AVAILABILITY

Lead contact

Further information and requests for resources and reagents should be directed to and will be fulfilled by the lead contact, Ezequiel Petrillo, petry1@gmail.com or petry@fbmc.fcen.uba.ar

Materials availability

- This study did not generate new unique reagents.

Data and code availability

- This paper analyzes existing, publicly available data. Accession numbers for the datasets are listed in the key resources table. Original data (RT-PCR and RT-qPCR results and western blot images) have been deposited at Mendeley and are publicly available as of the date of publication of this manuscript. The DOI is listed in the key resources table.
- This paper does not report original code.
- Any additional information required to reanalyze the data reported in this paper is available from the lead contact upon request.

EXPERIMENTAL MODEL AND SUBJECT DETAILS

Plant material and growth conditions

For most experiments, the *Arabidopsis thaliana* Col-0 ecotype was used as wild-type. Transgenic plants used in this study were the RNAi line of *TOR* (*TARGET OF RAPAMYCIN*) 35-7 (Deprost et al., 2007). Seeds were stratified for three days in the dark at 4°C and then germinated on Murashige and Skoog (MS) medium buffered (pH 5.7) with 2-(N-Morpholino) ethanesulfonic acid (MS-MES) and containing 1.5% agar. Plants were grown at a constant temperature of 23°C under fluorescent lamps emitting white light of an intensity of irradiance between 70 and 100 μmol photons / m^2 sec. Growth conditions different from these are indicated in the text and figure legends.

METHOD DETAILS

Pharmacological, sugar and low oxygen treatments

Subsequent to growing the plants for two weeks in constant light, they were incubated for 48 hours in the dark (Figure S1). For treatments with different compounds, plants on agar plates were submerged with 20 mL of liquid MS-MES medium supplemented with the drug or ethanol/dimethyl sulfoxide (vehicle) as a mock control. This was done after 47 hours of dark treatment (one hour before the end of the incubation in the dark). Vacuum was applied for five minutes to facilitate drug uptake by the different tissues. The used drugs and their final concentrations were: 15 μM DCMU (3-(3,4-dichlophenyl)-1,1-dimethylurea; Sigma); 20 μM 2,4-DNP (2,4-dinitrophenol; Sigma); and 2 or 20 μM AZD-8055 (Chemdea LLC). After the 47 + 1 hours of darkness (with/without drugs) the plants were incubated in light or dark for additional four hours. Sucrose was added alone or together with indicated drugs at the specified concentrations (see figure legends). Sorbitol was used as an osmotic control. Sucrose and sorbitol solutions were directly poured onto the agar plates to submerge the plants after 47 hours of darkness, letting the plants take up the sugars for one hour in the dark, or followed by the application of vacuum to facilitate the uptake of respective compounds by all tissues. After the end of the 48 hours of incubation in the dark, the plants were transferred to light and further incubated for four hours. Controls were kept in the dark.

Low oxygen treatments were carried out similarly but using isolated (detached) roots instead of seedlings. Briefly, after 47 hours of darkness, plants were dissected, and isolated roots were treated with sorbitol or sucrose (50 mM). After applying vacuum for five minutes, the atmosphere of the desiccation chamber used for vacuum infiltration was replaced with air saturated in nitrogen (from a liquid nitrogen canister). The incubation lasted four hours. Air exchange in the desiccation chamber ensured a constant environment of nitrogen saturation. For the control (normal oxygen level), ambient air was used instead of nitrogen saturated air.

For the glucose and Torin2 treatments: *A. thaliana* seedlings were grown to quiescence and then supplied with 15 mM glucose or with 15 mM glucose plus 5 μM Torin2 (Scarpin et al., 2020; Xiong et al., 2013). Two hours after the treatment, at least 600 seedlings for each treatment were collected and their RNA was extracted and used to construct RNA-Seq libraries (Project PRJNA639161). This experiment was repeated two more times to generate three replicates. Libraries were sequenced using the Illumina platform (Scarpin et al., 2020).

RT- PCRs for alternative splicing assessment

Extraction of plant total RNA was carried out using PeqGold TriFast (PeqLab) or TriPure (Sigma) following the manufacturer's instructions. For cDNA synthesis, 1 μg of RNA was used with the Reverse Transcription System (Promega) and oligo-dT as primer following the manufacturer's instructions. PCR amplification was performed with Phusion High-Fidelity DNA Polymerase (Thermo Fisher Scientific Inc.) using 2 μl of 1/5 diluted cDNA. The PCR program was: 1) 95°C x 3′, 2) 28-32 cycles of 95°C x 30," 58-60°C x 30," 72°C 1', 3) 72°C x 5′. RT-PCR products were electrophoresed and detected using RedSafe (iNtRON Biotechnology) dye and an ultraviolet transilluminator. The relative intensities of the bands were measured by densitometry using ImageJ. In particular cases, radioactive alpha-[32P]-dCTP was used to assess the alternative splicing changes as described before (Petrillo et al., 2014). Results were also validated using Real Time RT-qPCR for individual isoforms. See Table S6 for primer sequences.

RT-qPCR expression analysis

Synthesized cDNAs (above) were amplified with 1.5 U of Taq polymerase (Invitrogen) and SYBR Green (Roche) using the Eppendorf Mastercycler ep realplex. Primer sequences for RT-qPCR are available in Table S6.

Western blot

The organic phase of the PeqGold Trifast RNA isolation was used, and proteins were precipitated by addition of cold acetone and subsequent overnight incubation at −20°C. After centrifugation and ethanol washes of the protein pellets 100 μl of 1X Laemmli buffer were added. Ten μl of resuspended protein extract were loaded on SDS-PAGE gels. Primary antibodies were α-RPS6-phospho-S240 (1:5,000) (Dobrenel et al., 2016b) and α-RPS6 total (was generated similarly to RPS6-pS240 but using an unphosphorylated epitope (Dobrenel et al., 2016b)). HRP-conjugated goat anti-rabbit was used as secondary antibody (1:10,000-1:3000).

QUANTIFICATION AND STATISTICAL ANALYSIS

Statistics

Statistical analyses were carried out using InfoStat (https://www.infostat.com.ar/) 2018e. Same letters indicate means that are not statistically different ($p > 0.05$) for variance analyses with comparisons using Fisher LSD (Least Significant Difference) test from this package.

Statistical significance of the overlap between two groups of genes was calculated using the program at http://nemates.org/MA/progs/overlap_stats.html. The program calculates probability and a representation factor. A representation factor > 1 indicates more overlap than expected while a representation factor < 1 indicates less overlap than expected.

3D RNA-seq app analyses

This section of the Method details was adapted from the output "Results" of the 3D RNA-seq package (Guo et al., 2020).

The fastq files of the RNA-seq data were first analyzed online using Galaxy RNA-seq tools (https://usegalaxy.org), in particular "Salmon quant" that performs dual-phase, reads or mapping-based estimation of transcript abundance from RNA-seq reads (Galaxy Version 0.14.1.2). That was the input for the 3D RNA-seq App. This software was developed for rapid and accurate differential expression (DE), differential alternative splicing (DAS) gene and differential transcript usage (DTU) (3D) analysis (Calixto et al., 2018; Guo et al., 2020). The At-RTD2 database (https://ics.hutton.ac.uk/atRTD/) was used as input since it covers most of the known alternatively spliced isoforms in *A. thaliana* (Zhang et al., 2017). Using the loci (AGIs) as inputs at https://boxify.boku.ac.at/ it is possible to obtain sequences and schemes for all splicing variants represented in the At-RTD2 database. By adding primers listed in Table S6, the user can also obtain RT-PCR product sizes, sequences, and primer positions on the schemes of analyzed splicing variants.

The RNA-seq data of the root experiment (GSE132249) had four factor groups (Dark.X4d, Dark.X7d, Light.X4d and Light.X7d) and each had two biological replicates (eight samples in total). The RNA-seq data for the acute light experiment (GSE68560) had two factor groups (Dark, Light) and each had three biological replicates (six samples in total).

Read counts and transcript per million reads (TPMs) were generated using tximport R package version 1.10.0 and lengthScaledTPM method (Soneson et al., 2015) with inputs of transcript quantifications from tool salmon (Patro et al., 2017). Low expressed transcripts and genes were filtered based on analyzing the data mean-variance trend. The expected decreasing trend between data mean and variance was observed when expressed transcripts were determined as which had ≥ 1 of the eight samples with count per million reads (CPM) ≥ 1, which provided an optimal filter of low expression. A gene was expressed if any of its transcripts with the above criteria was expressed. The TMM method was used to normalize the gene and transcript read counts to *log*2-CPM (Bullard et al., 2010). The principal component analysis (PCA) plot showed the RNA-seq data did not have distinct batch effects allowing for further direct analysis of data. The Limma R package was used for 3D expression comparison (Law et al., 2014; Ritchie et al., 2015). To compare the expression changes between conditions of experimental design, the contrast groups were set as Light.X7d-Dark.X7d, Light.X4d-Dark.X4d, Dark.X7d-Dark.X4d, Light.X7d-Light.X4d in the case of Root experiment, as Light-Dark for the acute light treatment experiment, and as Glc-Glc.torin2 for the inhibitor RNA-seq experiment. For DE genes/transcripts, the *log*2 fold change (*L2FC*) of gene/transcript abundance were calculated based on contrast groups and significance of expression changes were determined using t test. P values of multiple testing were adjusted with BH to correct false discovery rate (FDR) (Benjamini and Yekutieli, 2001). A gene/transcript was significantly DE in a contrast group if it had adjusted p value < 0.01 and *L2FC* ≥ 1. At the alternative splicing level, DTU transcripts were determined by comparing the *L2FC* of a transcript to the weighted average of *L2FCs* (weights were based on their standard deviation) of all remaining transcripts in the same gene. A transcript was determined as significant DTU if it had an adjusted p value < 0.01 and ΔPS ≥ 0.1. For DAS genes, each individual transcript *L2FC* were compared to gene level *L2FC*, which was calculated as the weighted average of *L2FCs* of all transcripts of the gene. Then p values of individual transcript comparison were summarized to a single gene level p value with F-test. A gene was significantly DAS in a contrast group if it had an adjusted p value < 0.01 and any of its transcript had a Δ Percent Spliced (ΔPS) ratio ≥ 0.1.

Venn Diagrams

Tables of significantly affected genes obtained from the 3D RNA-seq tool analyses were the input for *Venny* 2.1 (An interactive tool for comparing lists with Venn's diagrams, developed by Oliveros JC, https://bioinfogp.cnb.csic.es/tools/venny/index.html).

Gene Ontology classification

Gene ontology (GO) classification and GO term overrepresentation tests were performed using PANTHER software tools (v.15.0) available at http://pantherdb.org (Mi et al., 2019).

ADDITIONAL RESOURCES

Alternative splicing isoforms web tool

We created a webtool called boxify (https://boxify.boku.ac.at) that allows for the easy creation and customization of publication-ready isoform figures for *Arabidopsis thaliana* (based on the AtRTD2 transcriptome annotation: Zhang et al., 2017) and that can aid in the visualization and design of PCR primer pairs.

Cell Reports, Volume *36*

Supplemental information

Light regulates alternative splicing outcomes

via the TOR kinase pathway

Stefan Riegler, Lucas Servi, M. Regina Scarpin, Micaela A. Godoy Herz, María G. Kubaczka, Peter Venhuizen, Christian Meyer, Jacob O. Brunkard, Maria Kalyna, Andrea Barta, and Ezequiel Petrillo

Figure S1

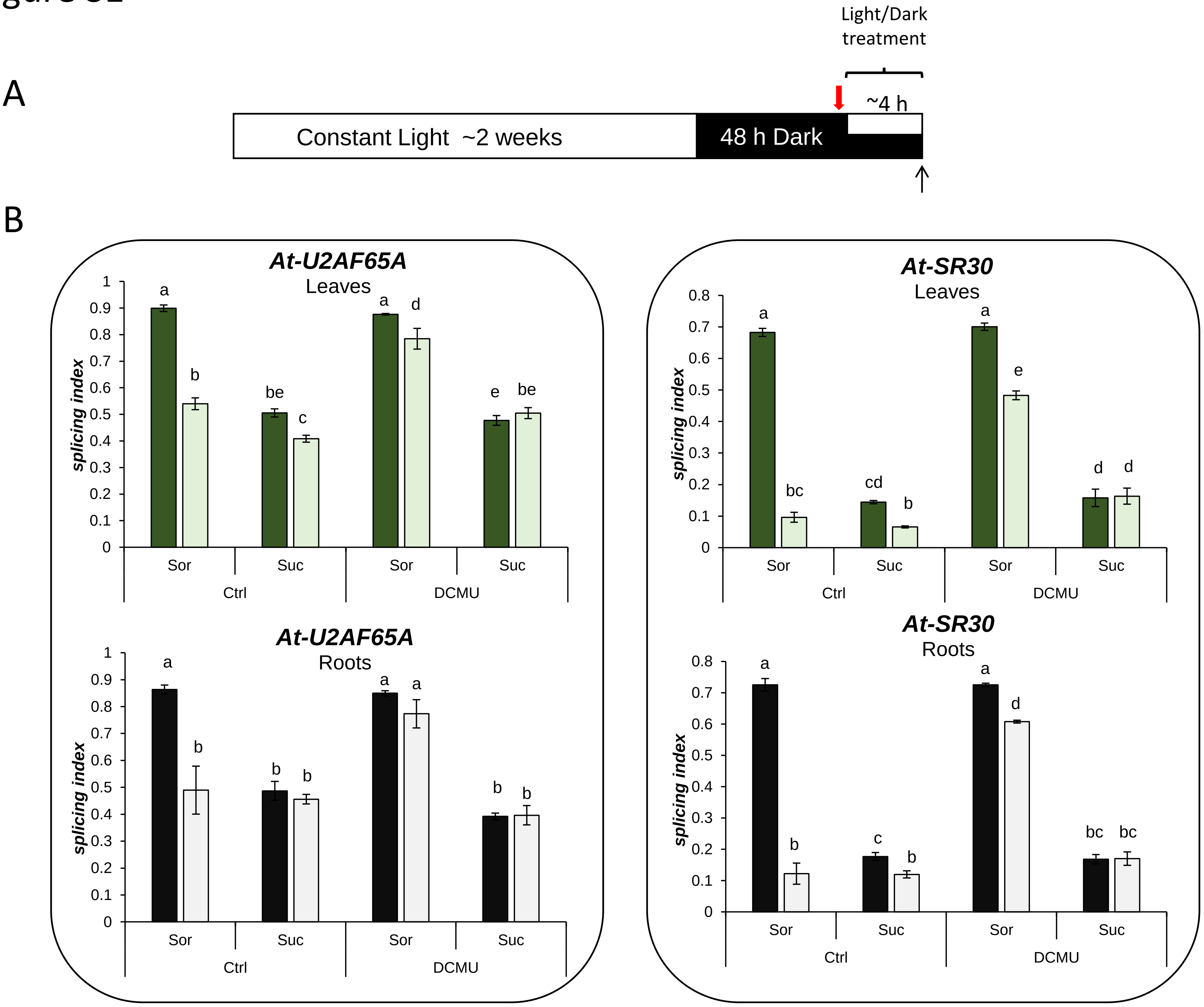

Figure S1. A) Standard treatment protocol. Seedlings were grown in Murashige and Skoog/2-(N-morpholino)ethanesulfonic acid buffered (MS-MES) agar plates (~15 seedlings per 10 cm Ø plate) for a period of two weeks and then transferred for 48 hours to darkness. After this period, seedlings were either transferred to light or kept in darkness for, approximately, additional four hours (light/dark treatment). Sorbitol, sucrose and the used drugs, were added in liquid media (20 mL) on top of the agar-growth media (20 mL) and vacuum was applied in order to ensure equal uptake by the different plant tissues. These compounds were added one hour before the end of the 48 hour darkness period (red arrow). Sample collection was performed at the end of the light/dark treatment and it is shown by the black arrow. When needed, prior sample collection, plants were dissected and leaves and roots were collected separatedly (post-treatment dissection). In particular experiments, plants were dissected before light/dark treatments (pre-treatment dissection). Related to Figures 1-3. **B) Sucrose effects on alternative splicing do not involve chloroplasts.** Alternative splicing changes are shown for *At-U2AF65A* (left) and *At-SR30* (right). *A. thaliana* seedlings were grown on MS-MES agar plates (~15 seeds per plate) for a period of two weeks under constant light, then transferred to darkness for 48 hours. Sorbitol (Sor, 100 mM) or sucrose (Suc, 100 mM) supplemented liquid media, with DCMU or without it (ethanol was used as control, Ctrl), were added on top of agar media one hour before the end of the 48 hours darkness period. Vacuum infiltration was applied for five minutes to increase the uptake of the different compounds by all the tissues. After the light (lighter bars) / dark (darker bars) treatments (~4h), leaves (green bars) and roots (grey bars) were dissected for sample collection. The graphs show splicing index means ± standard error (n=4). Same letters indicate means that are not statistically different ($p>0.05$). Statistics were done using InfoStat with Fisher LSD for comparisons. Related to Figures 1G-H.

Figure S2

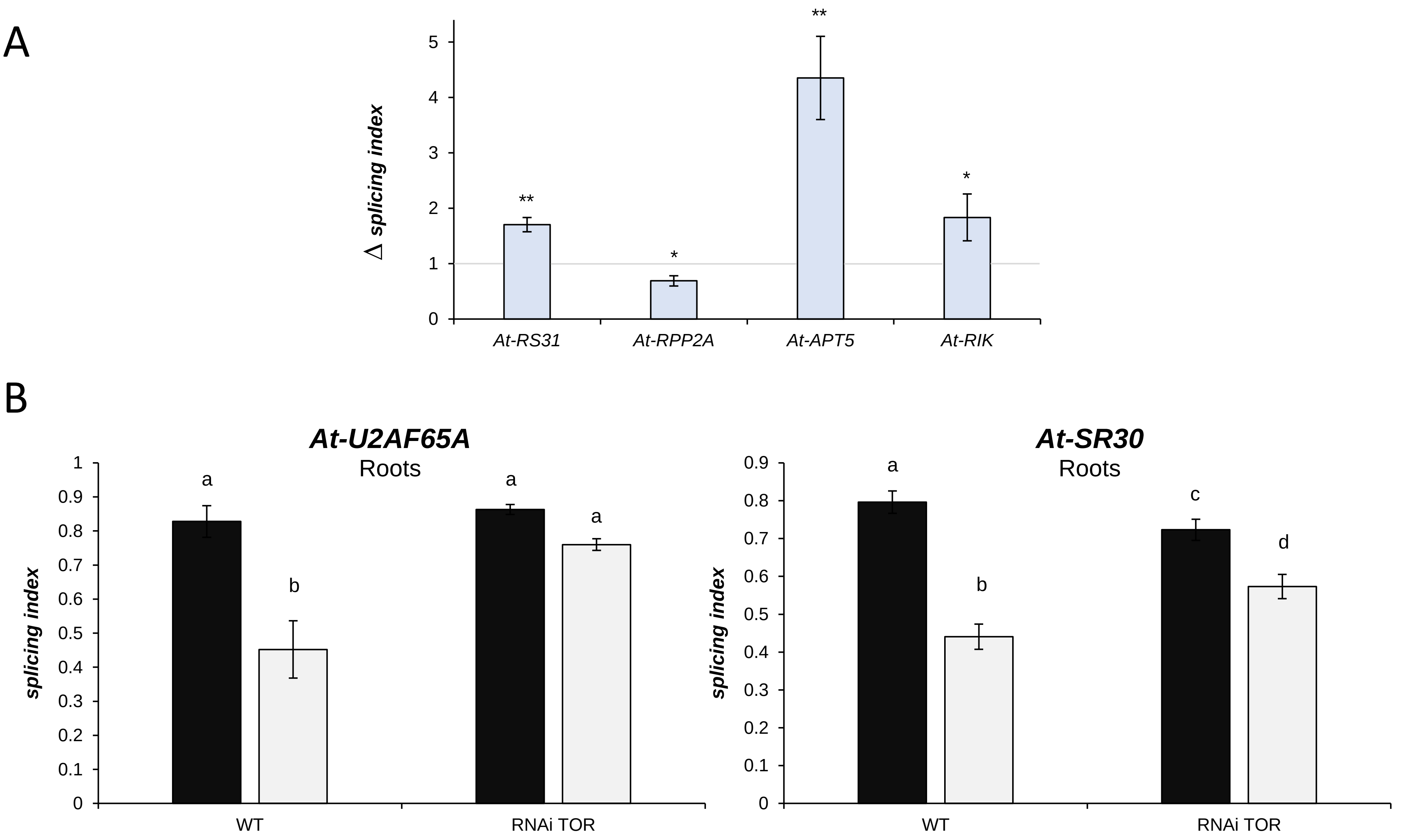

Figure S2. TOR regulates alternative splicing. A) Modulating TOR activity impacts alternative splicing of *At-RS31*, *RPP2A*, *APT5* and *RIK*. *A. thaliana* seedlings were grown on MS-MES agar plates (~15 seeds per plate) to quiescence and then supplied with either 15 mM glucose (control) or 15 mM glucose + 5 µM Torin2 (treatment). Following RT-PCR, splicing indexes were measured in both conditions and then related according to the following: Δ splicing index = splicing index (treatment) / splicing index (control). The graphs show Δ splicing index means ± standard deviation (n=3). Statistical significance was assessed via Student's t-test by comparing measured values with values drawn from normal distributions of mean = 1 (no change of splicing index upon treatment) with the same standard deviation as in the respective measured data. Student's t-test: ** $P < 0.01$; * $P < 0.05$. Related to Figure 2. **B) A transgenic RNAi knockdown line of TOR (RNAi TOR) shows disrupted responses to light on the splicing regulation in roots.** Alternative splicing changes are shown for *At-U2AF65A* and *At-SR30. A. thaliana* wild type (WT) and transgenic (35-7 TOR RNAi) line seedlings were grown on MS-MES agar plates (~15 seeds per plate) for a period of two weeks under constant light, then transferred to darkness for 48 hours. After light (lighter bars) / dark (darker bars) treatments for additional four hours, roots were dissected for sample collection. The graphs show splicing index means ± standard error (n=4). Same letters indicate means that are not statistically different (p>0.05). Statistical analyses were done using InfoStat with Fisher LSD for comparisons. Related to Figures 2B and 2D.

Figure S3

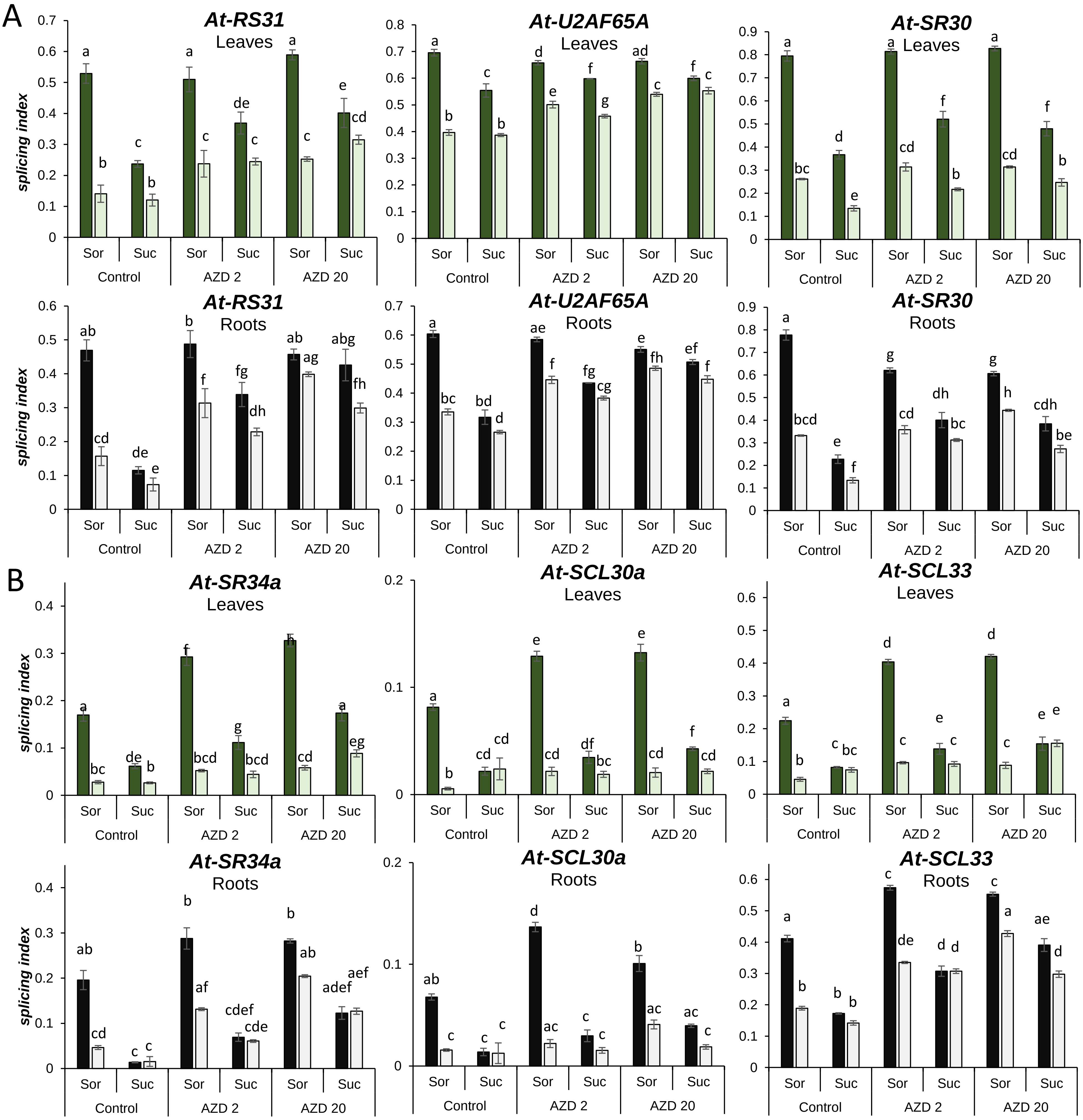

Figure S3: TOR kinase inhibition disrupts light and sucrose regulation of *At-RS31, At-U2AF65A, At-SR30* and *At-SR34a* but it has minor effects on *At-SCL30a* and *At-SCL33* alternative splicing events in roots. A-B) Alternative splicing changes are shown for *At-RS31* (**A,** left), *At-U2AF65A* (**A,** middle), *At-SR30* (**A,** right), *At-SR34a* (**B,** left), *At-SCL30a* (**B,** middle) and *At-SCL33* (**C,** right)*.* Upper panels are leaves and bottom panels roots. *A. thaliana* seedlings were grown on MS-MES agar plates (~15 seeds per plate) for a period of two weeks under constant light, then transferred to darkness for 48 hours. Sorbitol (Sor, 100 mM) or sucrose (Suc, 100 mM) supplemented liquid media, with two concentrations of AZD-8055 2 µM (AZD 2) and 20 µM (AZD 20) or without it (DMSO was used as control, Ctrl). Compounds were added in solution on top of agar media one hour before the end of the 48 hours darkness period. Vacuum infiltration was applied for five minutes to increase the uptake by all the tissues. After the light (lighter bars) / dark (darker bars) treatments (~4h), leaves (green bars) and roots (grey bars) were dissected for sample collection. The graphs show splicing index means ± standard error (n=3). Same letters indicate means that are not statistically different (p>0.05). Statistics were done using InfoStat with Fisher LSD for comparisons. Related to Figures 2E-F.

Figure S4

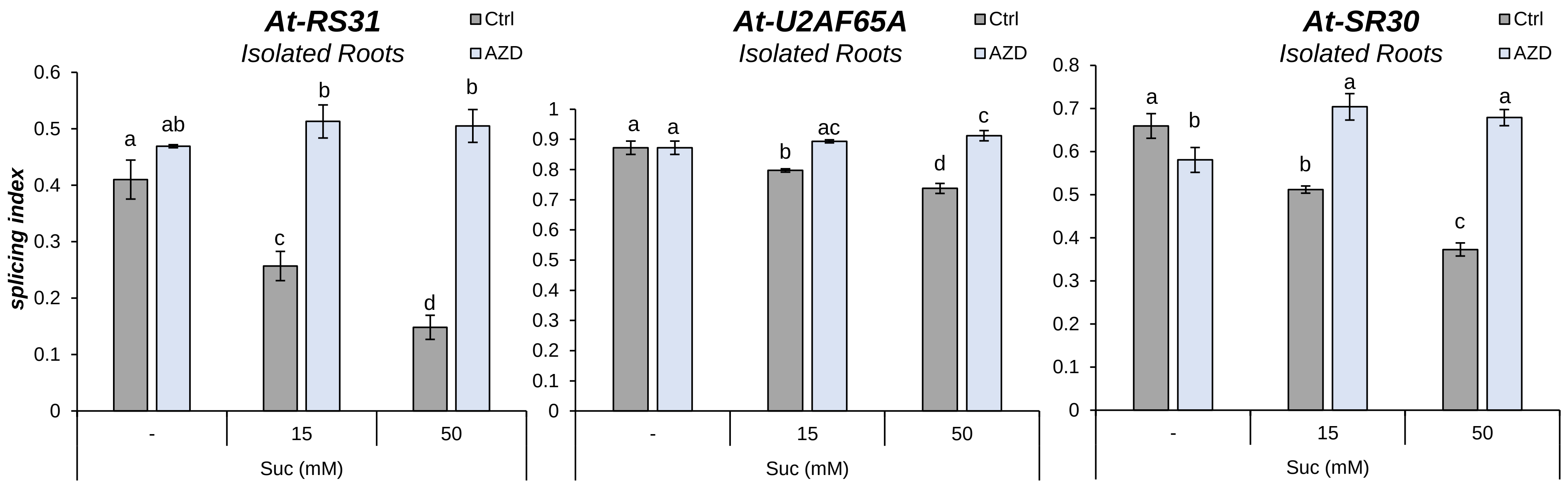

Figure S4. TOR kinase activity is necessary for isolated (detached) roots to change the alternative splicing of *At-RS31, At-U2AF65A* and *At-SR30* in response to sucrose. Sucrose (Suc) addition mimics light effects on alternative splicing, reducing the splicing index values in isolated roots in a dose dependent manner. The inhibition of TOR kinase activity by AZD-8055 (AZD) abolishes the effect of sucrose. *A. thaliana* plants were grown on MS-MES agar plates (~15 plants per plate) for a period of two weeks under constant light and then incubated in the dark for 48 hours. Roots were detached and transferred to 6-well plates with liquid media supplemented with sucrose 0, 15 or 50 mM. Sorbitol was used as osmotic control (to reach a total 50 mM concentration, together with sucrose, in every treatment). Twenty µM AZD-8055 (AZD, light blue bars) was used for treatments and dimethyl sulfoxide was used as control (Ctrl, grey bars). Vacuum was applied for five minutes to increase the uptake of the different compounds. The graphs show splicing index means ± standard error (n=3). Same letters indicate means that are not statistically different (p>0.05). Statistical analyses were done using InfoStat with Fisher LSD for comparisons. Related to Figures 2C-F.

Figure S5

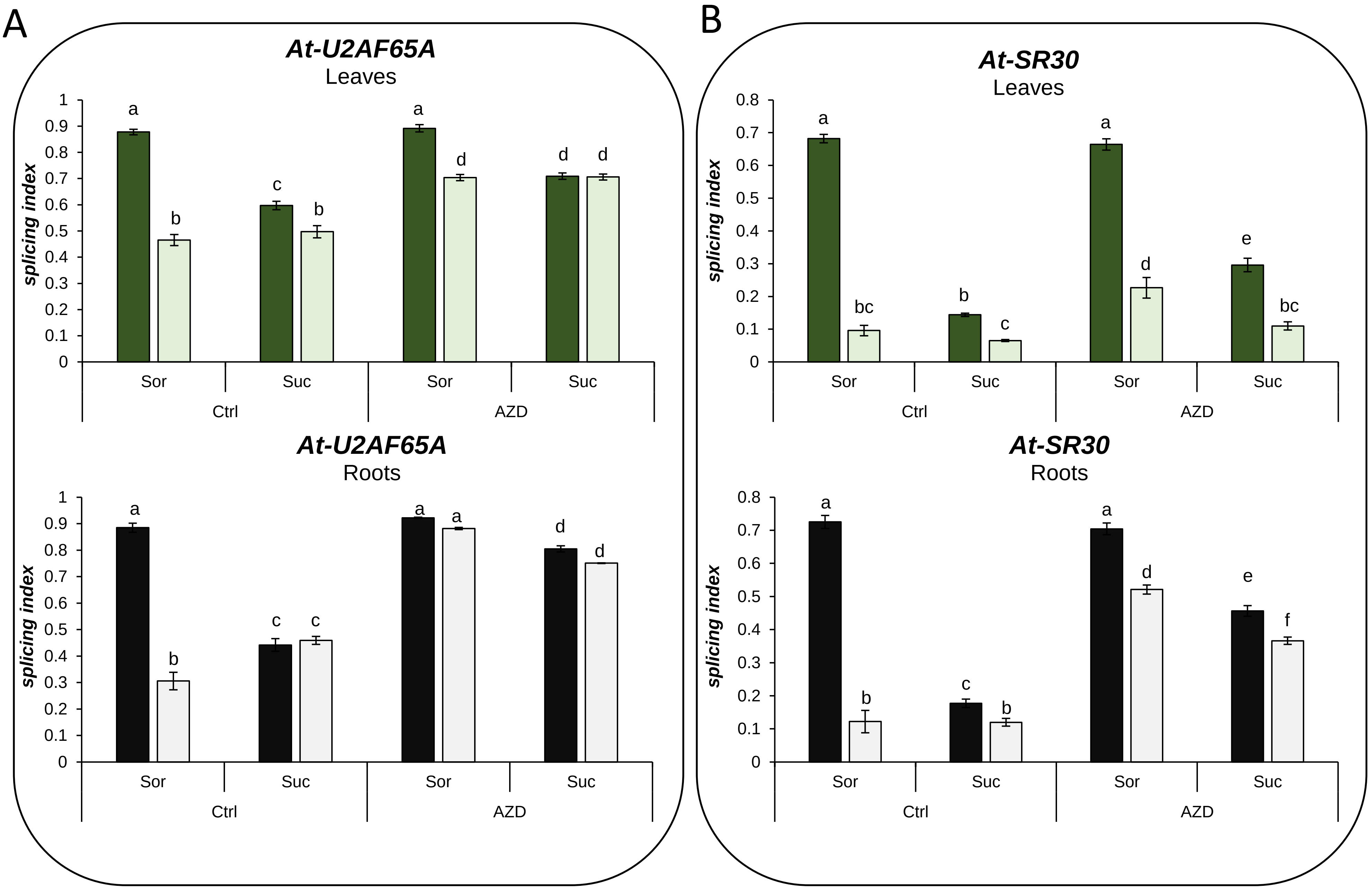

Figure S5. The inhibition of TOR kinase activity by AZD-8055 disrupts alternative splicing responses in roots. Alternative splicing changes are shown for (**A**) *At-U2AF65A* and (**B**) *At-SR30. A. thaliana* seedlings were grown on MS-MES agar plates (~15 seeds per plate) for a period of two weeks under constant light, then transferred to darkness for 48 hours. Sorbitol (Sor, 100 mM) or sucrose (Suc, 100 mM) supplemented liquid media, with 20 µM AZD-8055 (AZD) or without it (dimethyl sulfoxide as control, Ctrl), were added on top of the agar media one hour before the end of the 48 hours darkness period. Vacuum infiltration was applied for five minutes to increase the uptake of the different compounds by all the tissues. After the light (lighter bars) / dark (darker bars) treatments for additional four hours, leaves and roots were dissected for sample collection. The graphs show splicing index means ± standard error (n=4). Same letters indicate means that are not statistically different ($p>0.05$). Statistical analyses were done using InfoStat with Fisher LSD for comparisons. Related to Figures 2E-F, TOR kinase activity (RPS6-P/RPS6) can be seen there.

Figure S6

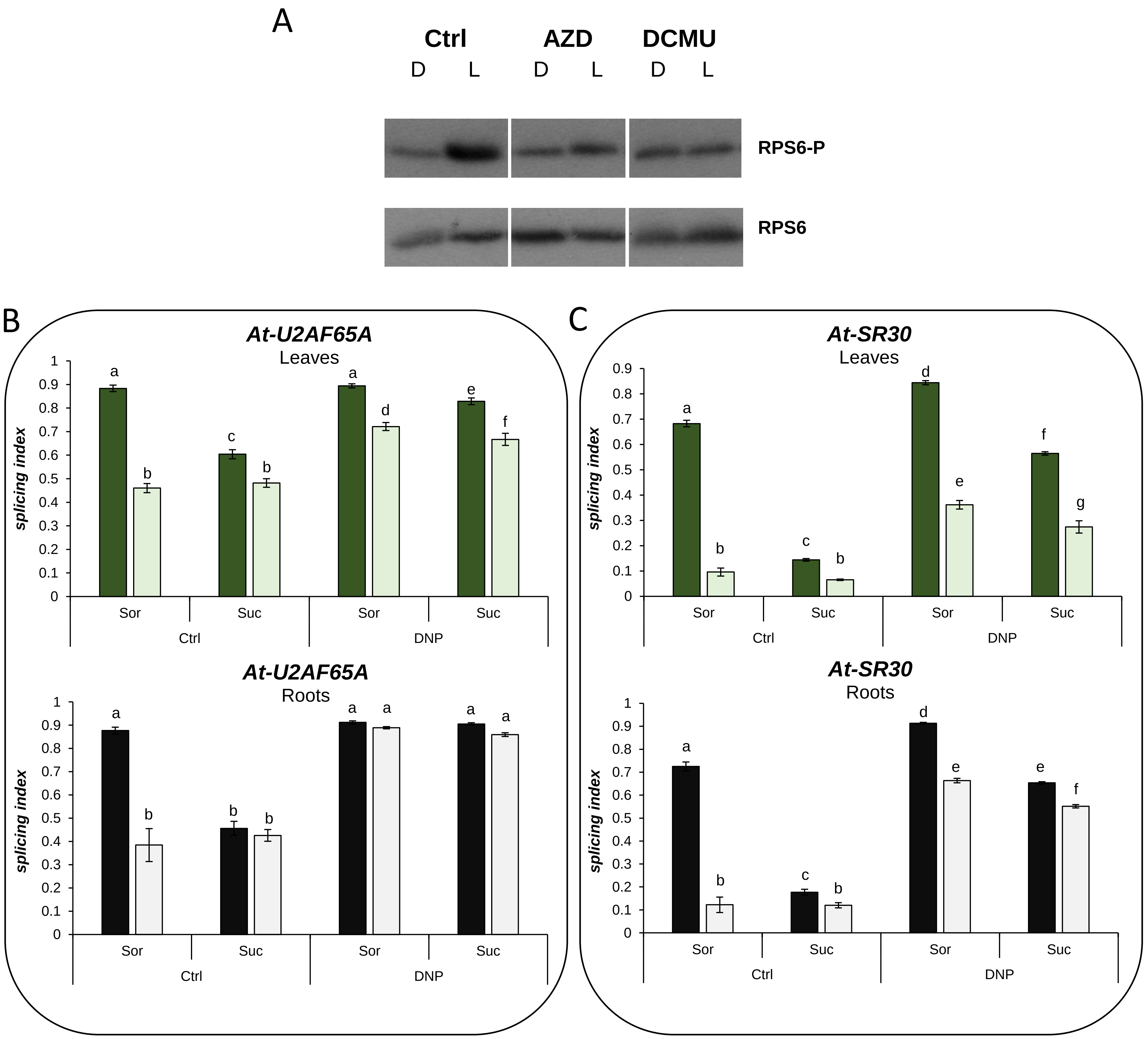

Figure S6. A) TOR kinase activity is modulated by the photosynthetic electron transport. Light (L) activates TOR kinase as revealed by the increase in the phosphorylation of RPS6 (RPS6-P) in light vs. dark (D). The inhibition of TOR kinase activity by AZD-8055 (AZD) diminishes the effect of light while blocking the electron transport with DCMU completely abolishes light induced phosphorylation of RPS6. Western blots to detect RPS6 phosphorylation and total levels in leaves after light/dark treatment in the presence of DCMU (15 µM) or AZD-8055 (20 µM). *A. thaliana* plants were grown on MS-MES agar plates (~15 plants per plate) for a period of two weeks under constant light and then incubated in the dark for 48 hours. Ethanol (for DCMU) and dimethyl sulfoxide (for AZD) were used combined as control (Ctrl) and supplemented to the same amounts in all the treatments. Vacuum was applied for five minutes to increase the uptake of the different compounds. Related to Figures 1G-H and 2C-D. **B-C) Proton gradients disruption by an uncoupler obliterates alternative splicing changes induced by light and sucrose in roots.** Alternative splicing changes are shown for (**B**) *At-U2AF65A* and (**C**) *At-SR30. A. thaliana* seedlings were grown on MS-MES agar plates (~15 seeds per plate) for a period of two weeks under constant light, then transferred to darkness for 48 hours. Sorbitol (Sor, 100 mM) or sucrose (Suc, 100 mM) supplemented media, with 20 µM DNP (dinitrophenol) or without it (ethanol was used as control, Ctrl), were added on top of the agar media one hour before the end of the 48 hours darkness period. Vacuum infiltration was applied for five minutes to increase the uptake of the different compounds by all the tissues. After light (lighter bars) / dark (darker bars) treatments for additional four hours, leaves and roots were dissected for sample collection. The graphs show splicing index means ± standard error (n=4). Same letters indicate means that are not statistically different ($p>0.05$). Statistical analyses were done using InfoStat with Fisher LSD for comparisons. Related to Figures 3A-B, TOR kinase activity (RPS6-P/RPS6) can be seen there.
